# Associations between day of admission, admission hyponatremia and hospital outcomes in medical patients: A retrospective multicenter cohort study

**DOI:** 10.1371/journal.pone.0335248

**Published:** 2025-10-27

**Authors:** Rajkumar Rajendram, Abdullah Abdulrahman AlShamrani, Ali Muhammad Alqaraishi

**Affiliations:** 1 Medical Protocol Department, King Abdulaziz Medical City, Ministry of National Guard – Health Affairs, Riyadh, Central Region, Saudi Arabia; 2 Research Unit, Department of Medical Education, College of Medicine, King Saud bin Abdulaziz University for Health Sciences, Riyadh, Central Region, Saudi Arabia; 3 Research Offices, King Abdullah International Medical Research Center, Riyadh, Central Region, Saudi Arabia,; 4 Department of Medicine, King Abdulaziz Medical City, Ministry of National Guard – Health Affairs, Riyadh, Central Region, Saudi Arabia; KPC Medical College and Hospital, INDIA

## Abstract

**Introduction:**

We investigated the relationship between admission day, serum sodium concentration, and outcomes in medical inpatients. Hyponatremia, the most common electrolyte abnormality in hospitalized adults, is associated with prolonged hospital length of stay (LOS) and higher mortality. Weekend admissions are also linked to worse outcomes, but the magnitude of this “weekend effect” may vary with diagnoses and particular day of admission.

**Design:**

Retrospective multicenter cohort study.

**Setting:**

Four Ministry of National Guard – Health Affairs hospitals in Saudi Arabia (January 1, 2016 – May 9, 2022).

**Participants:**

43,361 adult medical admissions. Patients with hypernatremia (n = 1,892) or LOS > 30 days (n = 2,988) were excluded.

**Interventions:**

Admissions were categorized by admission day (Sunday – Saturday) and serum sodium concentration (normonatremia and hyponatremia with severity sub-classification).

**Main outcome measures:**

LOS, intensive therapy unit (ITU) admission, and mortality.

**Statistical analyses:**

Multivariable gamma generalized linear models (GLM) were used to evaluate LOS, and logistic regression for ITU admission and mortality, adjusting for age, sex, and hospital site. Sensitivity analyses examined associations with the COVID-19 pandemic.

**Results:**

Hyponatremia on admission (42.4%) was associated with longer LOS than normonatremia, on all admission days (except Fridays). Association strength between admission day and LOS decreased as the severity of hyponatremia increased. In Saudi Arabia the statutory weekend is Friday-Saturday. Friday admissions had the longest LOS, while Mondays had the shortest (reference = Sunday). Tuesday admissions showed an unexpectedly prolonged LOS, potentially reflecting internal medicine resident half day teaching activities. Mortality was not significantly associated with admission day or serum sodium concentration in adjusted analyses, but the study was underpowered to detect modest mortality differences (58% power for 10% relative risk reduction). Sensitivity analyses demonstrated that admission day associations with LOS and ITU admission remained consistent across the pre-COVID-19 and COVID-19 periods. Admission during the COVID-19 pandemic was associated with increased mortality (OR 1.41) in the primary analysis. Sensitivity analyses demonstrated that this excess mortality probably reflected an additive main effect of the COVID-19 period rather than a differential effect by day of admission, confirming that the primary findings on admission day associations are robust.

**Conclusion:**

This study reveals associations between admission day, serum sodium, and hospital outcomes. LOS varied with both admission day and hyponatremia severity. Friday admissions had the longest LOS, but admission day associations with LOS weakened with increasing hyponatremia severity. Institutional scheduling and staffing patterns may create day-specific variations that extend beyond the traditional weekend-weekday dichotomy. These temporal variations highlight opportunities for targeted workflow adjustments and resource allocation that could improve care delivery, and potentially reduce morbidity and mortality.

## Introduction

Hyponatremia, defined as a serum sodium concentration below 135 mmol/L, is one of the most common electrolyte abnormalities affecting up to 30% of hospitalized patients [1,2]. It is linked to a range of adverse outcomes, including increased mortality, prolonged hospital stays, and higher healthcare costs [3–5].

A well-documented phenomenon, the “weekend effect,” suggests that patients admitted to hospital on weekends may experience worse outcomes compared to those admitted during weekdays [6–9]. While studies have consistently shown higher weekend mortality [6–9], findings on the impact of admission day on length of stay (LOS) have been inconsistent. However, some studies found that the unadjusted LOS of weekend admissions was shorter [8], potentially reflecting higher early mortality rate in this group.

The mechanisms driving the “weekend effect” are complex, multifactorial and not fully understood [6,7]. Reduced staffing and resource availability on weekends are likely contributors, but patient-level factors, including illness severity also contribute [6,7]. On weekends, hospitals often have fewer senior medical staff and limited access to specialist diagnostic or ancillary services. This lack of full support can delay critical evaluations, slow treatment changes, and hinder proactive management of evolving clinical situations.

In Saudi Arabia, the statutory weekend comprises Friday and Saturday, creating a distinct temporal pattern of staffing and service availability compared with Western settings where Saturday‑Sunday constitute the weekend, offering a unique context to examine the phenomenon.

The weekend effect may be diagnosis-specific [6,7]. Conditions requiring swift, precise diagnosis, specialist laboratory tests, or rapid treatment are particularly vulnerable to resource limitations. Hyponatremia, especially when severe or acute in onset, requires immediate, precise clinical decisions, frequent sodium monitoring, and careful adjustment of fluids or medications to prevent complications like osmotic demyelination or cerebral oedema [1,2].

Weekend resource limitations delaying diagnosis, sodium re-evaluation, or appropriate treatment can exacerbate the complications of hyponatremia and worsen the outcomes of these vulnerable patients. Thus, the weekend effect could impact hyponatremia patients disproportionately given the time-sensitive and resource-dependent nature of its optimal management.

Previous studies have attempted to adjust for illness severity in weekend admissions. For example, factoring in serum sodium concentrations accounts for some of the mortality associated with weekend admissions [6]. The intricate relationship between serum sodium concentrations, specific admission day, patient outcomes (e.g., LOS) and complex markers of disease severity (e.g., the need for admission to an intensive therapy unit (ITU) or severity of hyponatremia) remains insufficiently well described in the existing literature.

Though first described in 2001 [9], exploring, understanding and mitigating the weekend effect remain pressing patient safety issues. Few studies have simultaneously examined the interplay between hyponatremia, day-of-week of admission, and patient outcomes, leaving the combined impact of these variables largely unexplored. This study addresses this gap by investigating the associations of admission day with outcomes in hospitalized patients with hyponatremia, specifically focusing on LOS, mortality, and ITU admission. Understanding these associations and potential interactions can inform targeted interventions to enhance patient care and optimize resource utilization.

### Aim

This study examines the associations between admission day, serum sodium concentration, and patient outcomes in hospitalized medical inpatients.

## Methods

### Data source

This study used electronic health records from four Ministry of National Guard – Health Affairs (MNGHA) hospitals in Saudi Arabia. The study period spanned January 1, 2016, to May 9, 2022. The MNGHA provides comprehensive, state-funded healthcare to National Guard employees and their dependents, including primary, secondary and tertiary care. While eligibility criteria exist, treatment for life threatening conditions is provided to all patients presenting to the emergency department. Ethical approval (IRB/2541/22) for this study (NCC22R/495/10) was obtained from the institutional review board (IRB) of King Abdullah International Medical Research Center (KAIMRC), MHNGA, Riyadh, Saudi Arabia. Data were accessed for research on December 28, 2022. The authors did not have access to information that could identify individual participants during or after data collection.

### Study design and participants

This retrospective, observational, multicenter cohort study analysed all adult (≥18 years) medical admissions within the study period (43361).

To limit the effect of outliers and focus on acute inpatient outcomes, we excluded admissions exceeding 30 days (2988). This cut-off focused the analysis on acute medical events, reducing the impact of chronic hospitalizations or transfers to other facilities that might obscure the direct effects of admission day on immediate outcomes. Furthermore, this approach aligns with common practice in acute care research, where prolonged stays often introduce confounding factors not directly related to initial admission circumstances.

Admission episodes lacking serum sodium concentration data were also excluded (7). Patients with hypernatremia (sodium >145 mmol/L; 1892) were retained in the baseline demographic analyses for descriptive purposes only and excluded from all subsequent outcome analyses. Re-admissions during the study period were treated as independent observations and included in the analysis.

### Staff education about weekend effects and electrolyte abnormalities

All staff receive standard institutional induction covering hospital policies and clinical workflows, without specific education about weekend effects or admission-day variations. Internal medicine residents receive structured teaching on electrolyte disorders, including hyponatremia management, during their training rotations. Nursing staff receive generic guidance on laboratory monitoring protocols. No targeted interventions addressing weekend admission patterns existed during the study period.

### Institutional awareness of weekend effects

Following preliminary analysis of these data and presentations at departmental meetings, institutional leadership implemented several interventions: (i) appointment of Hospital Medicine Fellows providing senior oversight during reduced-staffing periods; (ii) mandatory daily 17:00 internal medicine consultant ward rounds in the emergency department ensuring timely review of all admissions; (iii) twice-daily post-discharge clinics strengthening care transitions. These interventions occurred after data collection and therefore do not influence the reported findings, but demonstrate institutional commitment to addressing identified disparities.

### Measurements

In addition to standard demographic data (age and sex), information on the day and date of admission to hospital, admission serum sodium concentration, ITU admissions, LOS from presentation to the hospital and inpatient mortality were collected from the hospitals’ electronic healthcare records. Admission episodes with missing data were excluded from the final analyses.

#### Definitions.

The established reference range for serum sodium concentration (i.e., normonatremia) is 135−145 mmol/L. Hyponatremia was defined as serum sodium below 135 mmol/L. The severity of hyponatremia was graded as mild (130–134.9 mmol/L), moderate (125–129.9 mmol/L) or severe (< 125 mmol/L) using the classification described by Spasovski et al., 2014 [2]. In Saudi Arabia the weekend encompasses Friday and Saturday, while weekdays extend from Sunday to Thursday. Given the inclusion of data from January 1, 2016, to May 9, 2022, a period encompassing the Coronavirus 2019 (COVID-19) pandemic, data from 1^st^ January 2016–29^th^ February 2020 (pre-COVID) were compared with data from 1^st^ March 2020–9^th^ May 2022 (the approximate duration of significant COVID-19 pandemic impact).

### Study outcomes

The primary outcome was total hospital LOS (including the day of presentation to the emergency department). Length of stay was rounded up to a whole number of days prior to further analysis. Secondary outcomes included inpatient mortality and ITU admission. Admission to ITU was used as a as a secondary outcome measure and as a covariate in models for LOS and mortality, reflecting its role as both a marker of disease severity as well as an outcome of interest. Percentage of patients discharged before the following weekend was analyzed as an exploratory outcome.

Given the large sample size (>40,000 admissions) and continuous nature of LOS data, we anticipated adequate power for detecting clinically meaningful differences. Post-hoc power calculations confirmed >90% power to detect 0.5-day LOS differences between groups. Secondary outcomes (mortality, ITU admission) had lower event rates; with 2,428 deaths (6% mortality), we had approximately 58% power to detect a 10% relative mortality reduction, which we acknowledge as a study limitation.

### Statistical analysis

Baseline patient characteristics and clinical outcomes were summarized using descriptive statistics. Categorical data are presented as frequencies and percentages (%) and compared using the Chi-squared test. Odds ratios (OR) with 95% confidence intervals (CIs) were calculated for categorical comparisons. Continuous data are presented as means and standard deviations (SDs) if normally distributed, or as medians and interquartile ranges (IQR) if not normally distributed. Normality of continuous data was assessed using histograms and D’Agostino-Pearson tests. These revealed that LOS exhibited a right-skewed distribution. Thus, LOS data are also presented as median and interquartile range (IQR) and compared using Mann-Whitney U-tests or the Kruskal-Wallis test followed by Dunn’s test for pairwise comparisons.

Kaplan-Meier survival curves with 95%CIs were constructed to compare the cumulative incidence rate of all-cause mortality. To investigate the associations between patient characteristics and all prespecified clinical outcomes (LOS, ITU admission, and mortality), separate regression models were constructed for each dependent variable. All models were adjusted for potential confounders identified a priori based on clinical relevance and bivariate analyses, including age, sex, day-of-week on admission, serum sodium concentrations, COVID-19 pandemic period, and hospital site. The potential impact of day-of-week of admission was assessed using two approaches. First, day of admission dichotomized as weekday (coded as 0) versus weekend (coded as 1) to examine the overall weekend effect on LOS. Second, dummy coding was used with each day of the week from Monday to Saturday represented as a separate binary variable to allow assessment of day-specific variations with Sunday as reference.

As the LOS distribution was right-skewed, generalized linear models (GLMs) were used to investigate the associations between age, sex, day-of-week on admission, the need for ITU admission, serum sodium concentrations, and LOS. LOS was rounded to whole days and treated as count data. Accordingly, both a negative binomial GLM and a gamma GLM with a log link were fitted. Robust (sandwich) variance estimators were used for the Gamma GLM to mitigate heteroscedasticity. The Gamma GLM had better fit (lower Akaike Information Criterion (AIC)) than the Negative Binomial GLM. Multicollinearity among predictors was assessed using Generalized Variance Inflation Factors (GVIF), with all values remaining below 1.2, indicating no significant multicollinearity. For the Gamma GLM for LOS, the residual plots (e.g., Residuals vs Fitted and Scale-Location plots) revealed heteroscedasticity, and the ‘Q-Q Residuals’ plot indicated right-skew in the residuals’ upper tail.

To explore potential day-to-day differences, dummy coding was used for each day of the week from Monday to Saturday with Sunday serving as the reference category. This allowed assessment of the variation of LOS across specific weekdays. This dual approach enhances the analysis by capturing both the overall weekend effect and potential weekday-specific variations. The LOS model also included the need for ITU admission and inpatient mortality as co-variates to account for severity and outcome-related confounding.

For ITU admission and inpatient mortality, multivariable logistic regression analysis was performed to identify factors associated with these binary (Yes/No) dependent variables. The independent variables included age, sex, day-of-week on admission, and serum sodium concentrations. Results are presented as adjusted odds ratios (AOR) with 95%CIs. Model fit and assumptions for each regression model were rigorously assessed (e.g., residual plots for negative binomial regression; Hosmer-Lemeshow test for logistic regression).

Two approaches were used to assess the effect of the COVID-19 period. A main effects model was used to estimate the average COVID impact across all admission days. A sensitivity analysis including day×COVID interaction terms was then performed to test whether the COVID effect varied by admission day. In interaction models, the COVID main effect coefficient represents the effect for the reference day (Sunday), with other days’ effects modified by their respective interaction terms. Therefore, COVID effect estimates differ between models due to different parameterization rather than contradictory findings.

Statistical significance was defined as a two-sided P value <0.05. Bonferroni correction was applied for multiple pairwise comparisons. All data management processes and statistical analyses were performed using R statistical software (R Foundation for Statistical Computing, Vienna, Austria) and Excel (Office Version 2016, Microsoft, Redmond, USA) with the Real Statistics Resource Pack software (Release 8.9.1). Copyright (2013–2023) Charles Zaiontz. www.real-statistics.com [10].

While this methodology allows the identification of associations and differences between groups, this retrospective observational study design inherently limits the ability to infer causality. The observed associations may be influenced by unmeasured confounders, which cannot be fully accounted for in this type of analysis.

### Patient and public involvement

Patient and public involvement is not a common practice in this region. Therefore, patients were not involved in the study design or the selection of outcome measures.

## Results

This retrospective analysis of 43,361 admission episodes in 23,141 unique patients across four Saudi Arabian hospitals identified hyponatremia in 42.4% of admissions. Table 1 provides a detailed overview of the study population’s demographic characteristics and admission serum sodium concentrations. Table 2 summarizes the multicenter associations of admission day and serum sodium concentration with ITU admissions, mortality and LOS.

**Table 1:** Demographics, serum sodium on admission and outcomes of the whole study population stratified by hospital.

HOSPITAL	Riyadh	Medina	Dammam	Al Ahsa	All Hospitals
**DEMOGRAPHICS**					
**Total Admissions (N%)**	30243 (74.9%)	4254 (10.5%)	1930 (4.8%)	3939 (9.8%)	40366 (100%)
**Female (N%)**	15007 (37.2%)	2098 (5.2%)	1035 (2.6%)	2254 (5.6%)	20394 (50.5%)
**Male (N%)**	15236 (37.7%)	2156 (5.3%)	895 (2.2%)	1685 (4.2%)	19972 (49.5%)
**Age Years (mean±SD)**	63 ± 19.7	62.9 ± 19.4	55 ± 22.3	60.1 ± 20.9	62.3 ± 20
**Pre-COVID Admission**	19,472 (48.2%)	2,372 (5.9%)	1,164 (2.9%)	2,393 (5.9%)	25,401 (63%)
**SERUM SODIUM CONCENTRATION ON ADMISSION (N = 40366)**					
**<135 mmol/L (N%)**	12830 (31.8%)	1896 (4.7%)	610 (1.5%)	1789 (4.4%)	17125 (42.4%)
**135–145 mmol/L (N%)**	15910 (39.4%)	2205 (5.5%)	1238 (3.1%)	1996 (4.9%)	21349 (52.9%)
**>145 mmol/L (N%)**	1503 (3.7%)	153 (0.4%)	82 (0.2%)	154 (0.4%)	1892 (4.7%)
**Sodium mmol/L (mean±SD)**	134.7 ± 7.8	133.9 ± 7.7	135.9 ± 6.7	134.1 ± 7.6	134.6 ± 7.7
**OUTCOMES**					
**Length of stay** **Days (mean±SD)**	7.66 ± 6.0	7.46 ± 5.89	5.94 ± 4.82	7.39 ± 5.62	7.53 ± 5.91
**ITU admission (N%)**	5477 (59.1%)	1275 (13.8%)	617 (6.7%)	1899 (20.5%)	9268 (23.0%)
**Mortality (N%)**	1782 (73.4%)	290 (11.9%)	61 (2.5%)	295 (12.2%)	2428 (6.0%)

Legend. This table presents the demographics, serum sodium, intensive therapy unit (ITU) admissions and outcomes (length of stay, and mortality) of the study population stratified by hospital site. Data are presented as mean ± standard deviation (SD) or frequency and percentages (%) as appropriate. The sex distribution, admission serum sodium and COVID-19 admission strata are described as percentages of the total number of admissions (40,366). The means of age and serum sodium for each hospital were calculated using the number of admissions to each hospital. The percentages used to describe each hospital’s outcomes are presented with respect to the row totals. Thus, the percentages used to describe the whole cohort’s sex distribution and outcomes are presented with respect to the total number of admissions (40,366).

**Table 2:** Multicenter data on the association of day of admission and serum sodium with ITU admissions, mortality and length of stay.

Day		Admissions N; % (95%CI)			Sodium	ITU admissions N; % (95%CI)			Mortality N; % (95%CI)			Length of stay Days mean±SD, median (IQR)		
Serum Sodium		<135 mmol/L	135-145 mmol/L	>145 mmol/L	mmol/L mean±SD	<135 mmol/L	135-145 mmol/L	Odds Ratio (95%CI; p)	<135 mmol/L	135-145 mmol/L	Odds Ratio (95%CI; p)	<135 mmol/L	135-145 mmol/L	p
**Weekday**	**Sun**	2614 (42.3%)	3293 (53.3%)	275 (4.4%)	134.8 ± 7.6	576; 22% (20.4% to 23.6%)	689; 20.9% (19.5% to 22.3%)	1.07 (0.94 to 1.21; p = 0.3)	147; 5.6% (4.7% to 6.5%)	174; 5.3% (4.5% to 6%)	1.07 (0.85 to 1.34; p = 0.57)	7.41 ± 5.76 5 (3 –10)	6.74 ± 5.74 5 (3 –9)	p = 1.03x10^-11^*
	**Mon**	2533 (40.8%)	3383 (54.5%)	287 (4.6%)	134.8 ± 7.6	564; 22.3% (20.6% to 23.9%)	693; 20.5% (19.1% to 21.8%)	1.11 (0.98 to 1.26; p = 0.1)	126; 5% (4.1% to 5.8%)	163; 4.8% (4.1% to 5.5%)	1.03 (0.81 to 1.31; p = 0.78)	7.41 ± 5.82 5 (3–10)	6.72 ± 5.64 4 (3–9)	p = 2.45x10^-10^*
	**Tue**	2410 (40.1%)	3337 (55.5%)	269 (4.5%)	134.9 ± 7.5	555; 23% (21.3% to 24.7%)	707; 21.2% (19.8% to 22.6%)	1.11 (0.98 to 1.26; p = 0.1)	126; 5.2% (4.3% to 6.1%)	155; 4.6% (3.9% to 5.4%)	1.13 (0.89 to 1.44; p = 0.31)	7.93 ± 6 6 (3–10)	7.11 ± 5.66 5 (3–9)	p = 3.69x10^-10^*
	**Wed**	2485 (41.2%)	3263 (54.1%)	284 (4.7%)	134.8 ± 7.6	534; 21.5% (19.9% to 23.1%)	721; 22.1% (20.7% to 23.5%)	0.96 (0.85 to 1.1; p = 0.58)	131; 5.3% (4.4% to 6.2%)	171; 5.2% (4.5% to 6%)	1.01 (0.8 to 1.27; p = 0.96)	7.76 ± 5.87 6 (3–9)	7.14 ± 6.04 5 (3–9)	p = 4.3x10^-11^*
	**Thu**	2414 (43.4%)	2912 (52.4%)	234 (4.2%)	134.3 ± 7.5	587; 24.3% (22.6% to 26%)	699; 24% (22.5% to 25.6%)	1.02 (0.9 to 1.15; p = 0.79)	143; 5.9% (5% to 6.9%)	148; 5.1% (4.3% to 5.9%)	1.18 (0.93 to 1.49; p = 0.18)	7.88 ± 5.88 6 (4–10)	7.55 ± 6.04 6 (3–9)	p = 0.00029*
**Weekend**	**Fri**	2273 (44.6%)	2549 (50%)	279 (5.5%)	134.4 ± 8.2	492; 21.6% (20% to 23.3%)	640; 25.1% (23.4% to 26.8%)	0.82 (0.72 to 0.94; p = 0.005*)	123; 5.4% (4.5% to 6.3%)	137; 5.4% (4.5% to 6.3%)	1.01 (0.78 to 1.29; p = 0.96)	7.94 ± 5.87 6 (4–11)	7.77 ± 5.83 6 (4–11)	p = 0.15
	**Sat**	2396 (45.4%)	2612 (49.5%)	264 (5%)	134.3 ± 8.1	614; 25.6% (23.9% to 27.4%)	691; 26.5% (24.8% to 28.1%)	0.96 (0.84 to 1.09; p = 0.5)	158; 6.6% (5.6% to 7.6%)	152; 5.8% (4.9% to 6.7%)	1.14 (0.91 to 1.44; p = 0.26)	7.73 ± 5.72 6 (4–11)	7.08 ± 5.58 5 (3–9)	p = 1.89x10^-07^*
**Total**		17125 (42.4%)	21349 (52.9%)	1892 (4.7%)	134.6 ± 7.7	3922; 22.9% (22.3% to 23.5%)	4840; 22.7% (22.1% to 23.2%)	1.01 (0.97 to 1.06; p = 0.59)	954; 5.6% (5.2% to 5.9%)	1100; 5.2% (4.9% to 5.4%)	1.09 (0.99 to 1.19; p = 0.07)	7.72 ± 5.85 6 (4–10)	7.13 ± 5.8 5 (3–9)	P = 0.000*

Legend. This table compares the outcomes of patients admitted with hyponatremia (serum sodium <135 mmol/l) or normonatremia (serum sodium 135–145 mmol/L), stratified by admission day. In Saudi Arabia the weekend is Friday-Saturday, while Sunday to Thursday are weekdays. Data presented as mean±SD (standard deviation) and median and interquartile ranges (IQR) were compared using Mann-Whitney U-tests. Data on intensive therapy unit (ITU) admissions and mortality, presented as frequency and percentages with 95% confidence intervals (95%CI), were compared using the Chi-squared test. Statistically significant differences after the application of Bonferroni correction (p < 0.0071; i.e., 0.05/7) are indicated (*). The outcomes of patients stratified by admission day, serum sodium and hospital site are presented in S1 Table (ITU admission), S2 Table (mortality) and S3 Table (length of stay).

### Admission serum sodium and intensive therapy unit admissions

Descriptive analyses show variations in ITU admission rates stratified by admission day and serum sodium concentration (normonatremia (S4 Table) or hyponatremia (S5 Table)). Friday admissions with hyponatremia were significantly less likely to be admitted to an ITU during their inpatient stay than normonatremic admissions on the same day (p = 0.005; Table 2). Mildly hyponatremic admissions were more likely to be admitted to ITU than those with moderate or severe hyponatremia (Table 3; p = 0.043).

**Table 3:** Multicenter data showing the associations between admission day and severity of hyponatremia as well as ITU admissions, mortality and length of stay.

Day		Admissions N; %			ITU admissions N; % (95%CI)				Mortality N; % (95%CI)				Length of stay Days mean±SD median (IQR)			
Severity of Hyponatremia		Mild	Moderate	Severe	Mild	Moderate	Severe	p	Mild	Moderate	Severe	p	Mild	Moderate	Severe	P
**Weekday**	**Sun**	1535 (24.8%)	547 (8.8%)	532 (8.6%)	327; 21.3% (19.3% to 23.4%)	120; 21.9% (18.5% to 25.4%)	129; 24.2% (20.6% to 27.9%)	p = 0.37	76; 5% (3.9% to 6%)	31; 5.7% (3.7% to 7.6%)	40; 7.5% (5.3% to 9.8%)	p = 0.086	7.1 ± 5.6 5 (3 –9 )	7.7 ± 5.8 5 (3 –10 )	7.6 ± 5.7 5 (4 –10 )	p = 0.023*
	**Mon**	1477 (23.8%)	480 (7.7%)	576 (9.3%)	354; 24% (21.8% to 26.1%)	97; 20.2% (16.6% to 23.8%)	113; 19.6% (16.4% to 22.9%)	p = 0.05*	62; 4.2% (3.2% to 5.2%)	32; 6.7% (4.4% to 8.9%)	32; 5.6% (3.7% to 7.4%)	p = 0.074	8 ± 6.1 6 (3 –10)	7.7 ± 6.2 5 (3 –10 )	7.9 ± 6.1 5 (4-9.25)	p = 0.0040*
	**Tue**	1443 (24%)	443 (7.4%)	524 (8.7%)	351; 24.3% (22.1% to 26.5%)	88; 19.9% (16.1% to 23.6%)	116; 22.1% (18.6% to 25.7%)	p = 0.13	68; 4.7% (3.6% to 5.8%)	28; 6.3% (4.1% to 8.6%)	30; 5.7% (3.7% to 7.7%)	p = 0.35	7.7 ± 5.9 6 (3 –1 0)	8.1 ± 5.9 7 (4 –10 )	7.8 ± 5.8 6 (4 –9 )	p = 0.56
	**Wed**	1492 (24.7%)	491 (8.1%)	502 (8.3%)	328; 22% (19.9% to 24.1%)	99; 20.2% (16.6% to 23.7%)	107; 21.3% (17.7% to 24.9%)	p = 0.69	75; 5% (3.9% to 6.1%)	28; 5.7% (3.7% to 7.8%)	28; 5.6% (3.6% to 7.6%)	p = 0.80	7.6 ± 5.7 6 (4 –9)	8.2 ± 6.2 7 (4 –10 )	7.4 ± 5.5 6 (4 –9 )	p = 0.22
	**Thu**	1341 (24.1%)	513 (9.2%)	560 (10.1%)	330; 24.6% (22.3% to 26.9%)	133; 25.9% (22.1% to 29.7%)	124; 22.1% (18.7% to 25.6%)	p = 0.33	85; 6.3% (5% to 7.6%)	33; 6.4% (4.3% to 8.6%)	25; 4.5% (2.8% to 6.2%)	p = 0.25	8 ± 6.1 6 (4 –11)	8.7 ± 6.2 7 (4 –12)	7.8 ± 5.9 5 (4 –9 )	p = 0.00072*
**Weekend**	**Fri**	1276 (25%)	439 (8.6%)	558 (10.9%)	288; 22.6% (20.3% to 24.9%)	74; 16.9% (13.4% to 20.4%)	130; 23.3% (19.8% to 26.8%)	p = 0.024*	70; 5.5% (4.2% to 6.7%)	24; 5.5% (3.3% to 7.6%)	29; 5.2% (3.4% to 7%)	p = 0.97	7.6 ± 5.8 6 (4 –10)	7.9 ± 5.6 6 (4 –11)	7.8 ± 5.6 6 (4 –9)	p = 0.83
	**Sat**	1313 (24.9%)	517 (9.8%)	566 (10.7%)	352; 26.8% (24.4% to 29.2%)	140; 27.1% (23.2% to 30.9%)	122; 21.6% (18.2% to 24.9%)	p = 0.04*	69; 5.3% (4% to 6.5%)	41; 7.9% (5.6% to 10.3%)	48; 8.5% (6.2% to 10.8%)	p = 0.014*	7.2 ± 5.8 5 (3 –9)	8 ± 5.9 6 (4 –11)	7.7 ± 5.2 6 (4-10.75)	p = 0.11
**Total**		9877 (24.5%)	3430 (8.5%)	3818 (9.5%)	2330; 23.6% (22.8% to 24.4%)	751; 21.9% (20.5% to 23.3%)	841; 22% (20.7% to 23.3%)	p = 0.043*	505; 5.1% (4.7% to 5.5%)	217; 6.3% (5.5% to 7.1%)	232; 6.1% (5.3% to 6.8%)	p = 0.0086*	7.6 ± 5.9 6 (3 –10)	8 ± 6 6 (4 –11)	7.7 ± 5.7 6 (4 –9)	p = 0.0000015*

**Legend.** This table stratifies the outcomes of admission episodes with mild (130–134.9 mmol/L), moderate (125–129.9 mmol/L) or severe hyponatremia (< 125 mmol/L) by admission day. In Saudi Arabia the weekend is Friday-Saturday, while Sunday to Thursday are weekdays. Data on intensive therapy unit (ITU) admissions and mortality, presented as frequency and percentages with 95% confidence intervals (95%CI), were compared using the Chi-squared test. The data on length of stay (LOS), presented as mean±SD (standard deviation) and median and interquartile range (IQR) were compared using the Kruskal-Wallis test. Statistically significant differences (p < 0.05) are indicated (*). Further analysis of the variation in LOS with the severity of hyponatremia and admission day is presented in S8–S10 Tables. Kaplan-Meier analysis of the variation in mortality with the severity of hyponatremia is presented in Fig 1.

### Admission day and intensive therapy unit admission

**Fig 1:**
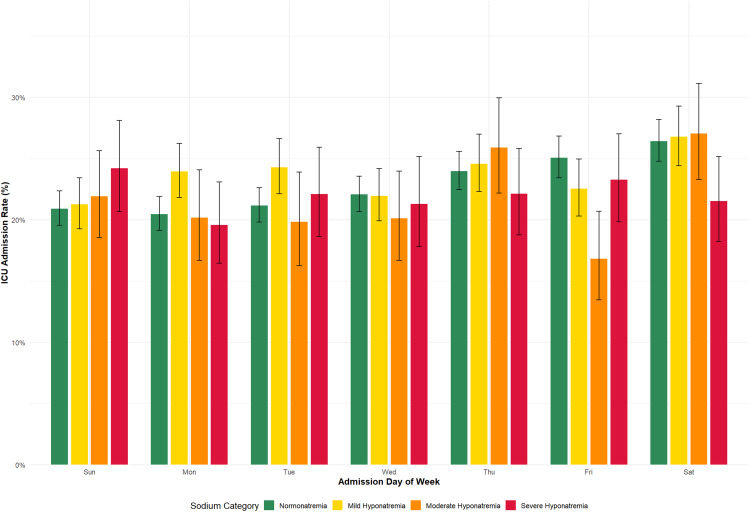
ITU Admission Rates by Admission Day and Serum Sodium Category. Legend. Bars represent the proportion of admissions admitted to the intensive therapy unit (ITU) according to day of hospital admission (Sunday through Saturday) and serum sodium category at presentation (normonatremia: 135–145 mmol/l, mild hyponatremia: 130–134.9 mmol/l, moderate hyponatremia: 125–129.9 mmol/l, severe hyponatremia: < 125 mmol/l). Error bars indicate 95% confidence intervals for each proportion. Patients with mild hyponatremia had the highest overall ITU admission rate (23.6%), while moderate and severe hyponatremia showed similar rates (21.9% and 22.0% respectively). ITU admission rates increased progressively from Sunday through Saturday across all sodium categories, with Saturday showing the highest admission rates.

The overall trends were similar between groups. A general increase in the risk of ITU admission was observed from Sunday to Saturday admissions in both normonatremic and hyponatremic cohorts. The need for ITU admission was highest for Saturday admissions in both cohorts. Patients admitted on Thursdays or weekends (Friday-Saturday), were more likely to be admitted to ITU than those admitted on other days (Sunday-Wednesday; Fig 1, Tables 2, S4 and S5).

### Inpatient mortality

**Fig 2:**
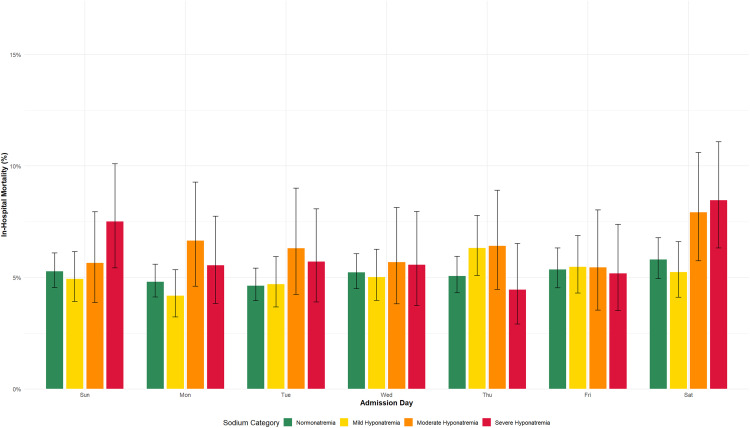
In-Hospital Mortality by Admission Day and Serum Sodium Category (Excluding Hypernatremia). Legend. Bars display the proportion of admissions who died during hospitalization according to day of admission (Sunday through Saturday) and serum sodium category at presentation (normonatremia: 135–145 mmol/l, mild hyponatremia: 130–134.9 mmol/l, moderate hyponatremia: 125–129.9 mmol/l, severe hyponatremia: < 125 mmol/l). Error bars represent 95% confidence intervals for each proportion. Mortality rates ranged from 4.8% to 6.6% across sodium categories and admission days, with no statistically significant differences after adjustment for confounders (Table 4).

**Fig 3:**
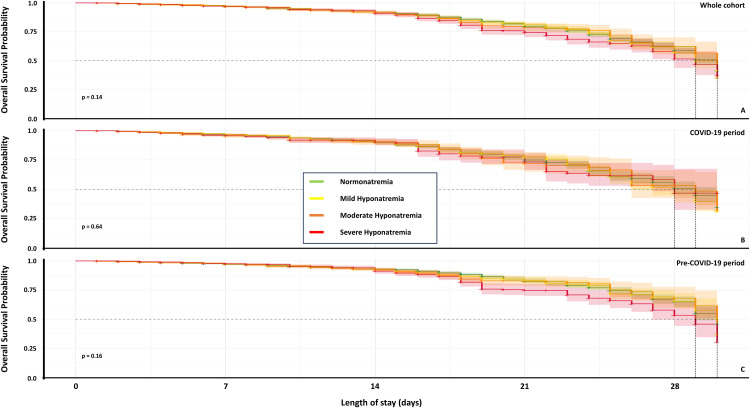
Thirty-day inpatient survival by admission serum sodium concentration: Kaplan-Meier analysis stratified by pre- and during-COVID-19 pandemic periods. Legend. These Kaplan-Meier analyses assessed the 30-day inpatient survival probability of admissions with normonatremia (135−145 mmol/L) or mild (130-134.9 mmol/L), moderate (125-129.9 mmol/L), or severe (<125 mmol/L) hyponatremia. A. Survival curves for the entire study cohort. B. Survival data for pre-COVID admissions (01/01/16-29/02/20). C. Survival data for admissions during the COVID-19 pandemic (01/03/20-09/05/22). No statistically significant differences were observed between groups. Log-rank test p values are shown. Shaded areas around each curve represent 95% confidence intervals.

Crude mortality rates were 5.2% in normonatremic admissions and 5.6% in hyponatremic admissions (p = 0.07; Table 2). Neither admission day nor serum sodium concentration significantly associated with mortality (Table 2; pairwise analyses not shown for brevity). Descriptive subgroup analyses revealed numerical differences in mortality between mild (5.1%), moderate (6.3%) and severe (6.1%) hyponatremia (χ^2^ 9.52; p = 0.009; Fig 2, Table 3). However, Kaplan-Meier survival curves showed no significant divergence (log rank test p = 0.15; Fig 3) and multivariable logistic regression confirmed no significant association between sodium severity and mortality after adjusting for age, sex, admission day, COVID-19 period, and hospital site (Table 4; p = 0.13). The discrepancy between crude and adjusted analyses may reflect confounding by age and comorbidity.

**Table 4:** Multivariable regression models for intensive therapy unit (ITU) admission, inpatient mortality and length of stay.

	ITU Admission		Mortality		Length of Stay	
Variable	Adjusted OR (95%CI)	p-value	Adjusted OR (95%CI)	p-value	Adjusted IRR (95%CI)	p-value
**Age**	1.00 (1.00 to 1.00)	0.011*	1.04 (1.03 to 1.04)	<0.001*	1.01 (1.01 to 1.01)	<0.001*
**Sex**		<0.001*		0.01*		0.38
Male	—		—		—	
Female	0.87 (0.83 to 0.92)		0.88 (0.80 to 0.97)		1.01 (0.99 to 1.02)	
**Admission day**		<0.001*		0.47		<0.001*
Sun	—		—		—	
Mon	1.00 (0.92 to 1.1)		0.88 (0.74 to 1.05)		1.00 (0.97 to 1.03)	
Tue	1.04 (0.95 to 1.14)		0.84 (0.71 to 1.00)		1.06 (1.03 to 1.09)	
Wed	1.05 (0.96 to 1.15)		0.92 (0.77 to 1.09)		1.04 (1.01 to 1.07)	
Thu	1.21 (1.11 to 1.33)		0.88 (0.74 to 1.05)		1.08 (1.05 to 1.11)	
Fri	1.17 (1.067 to 1.28)		0.87 (0.72 to 1.03)		1.10 (1.07 to 1.13)	
Sat	1.33 (1.21 to 1.46)		0.96 (0.81 to 1.14)		1.01 (0.98 to 1.04)	
**Serum sodium**		0.062		0.13		<0.001*
Linear	0.94 (0.88 to 1.00)		1.08 (0.96 to 1.2)		1.03 (1.01 to 1.05)	
Quadratic	0.98 (0.92 to 1.05)		1.00 (0.89 to 1.12)		0.95 (0.93 to 0.97)	
Cubic	1.06 (0.99 to 1.13)		0.88 (0.78 to 1.00)		0.98 (0.96 to 1.00)	
**COVID-19**		0.18		<0.001*		<0.001*
Pre-COVID-19	—		—		—	
COVID-19	0.97 (0.92 to 1.02)		1.41 (1.28 to 1.55)		1.69 (1.66 to 1.72)	
**ITU admission**	—	—		<0.001*		<0.001*
No	—	—	—		—	
Yes	—	—	11.9 (10.7 to 13.2)		1.20 (1.16 to 1.25)	
**Hospital**		<0.001*		<0.001*		0.014*
Riyadh	—		—		—	
Medina	1.92 (1.78 to 2.06)		0.78 (0.67 to 0.90)		0.99 (0.97 to 1.00)	
Dammam	2.18 (1.96 to 2.41)		0.41 (0.30 to 0.54)		0.75 (0.72 to 0.77)	
Al Ahsa	4.20 (3.91 to 4.51)		0.70 (0.60 to 0.80)		0.84 (0.82 to 0.86)	

Legend. Results of multivariable regression analysis examining the association between age, sex, admission day, serum sodium and admission to the intensive therapy unit (ITU), mortality or length of stay (LOS). Three models were constructed: logistic regression for ITU admission and inpatient mortality (presented as adjusted odds ratios (OR) with 95% confidence intervals (CI)) and gamma regression for LOS (presented as incidence rate ratios (IRR) with 95%CI). Dummy coding (one-hot encoding) was used for day-of-week variables using Sunday as the reference. In Saudi Arabia the weekend is Friday-Saturday, while Sunday to Thursday are weekdays. Male was the reference for sex. The pre-COVID-19 period was the reference for the COVID-19 period. P-values are based on two-tailed tests. Model fit statistics: ITU Admission model (N = 38,474; AIC = 39,537; BIC = 39,674). Mortality model (N = 38,474; AIC = 12,872; BIC = 13,018). LOS model (N = 38,474; AIC = 215,565; BIC = 215,728). Statistically significant differences (p < 0.05) are indicated (*). Abbreviations: AIC, Akaike Information Criterion; BIC, Bayesian Information Criterion; N, Number of Observations.

With 2,428 deaths among 40,366 admissions (mortality 6%), post‑hoc power calculations indicate 58% power to detect a 10% relative mortality reduction (α = 0.05). This limits the ability to detect clinically meaningful mortality differences, and the absence of significant associations should be interpreted cautiously. Larger prospective studies with comprehensive comorbidity measures are needed.

### Variation in length of stay with admission serum sodium

Table 2 presents the LOS stratified by admission and serum sodium concentration. Hyponatremic admissions consistently had a longer LOS than those with normonatremia regardless of admission day (except for Fridays). Independent of serum sodium, Friday admissions had the longest LOS (Tables 2, S4 and S5). However, the correlation between LOS and serum sodium was weak (r = 0.037). S1 Fig shows the variation in the percentage of admissions discharged before the next weekend stratified by serum sodium concentration and admission day.

### Admission day, severity of hyponatremia and length of stay

S6 and S7 Tables stratify the LOS of admissions with normonatremia and hyponatremia by admission day. The Kruskal-Wallis test revealed statistically significant differences in LOS between at least two admission day groups in each cohort (normonatremia H(6)=157, p = 1.28x10^-31^; hyponatremia H(6)=49, p = 5.48x10^-09^).

**Fig 4:**
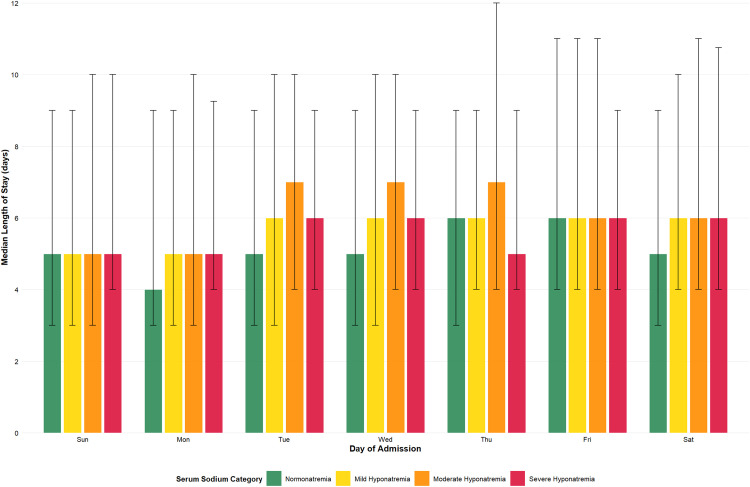
Median Length of Stay by Day of Admission and Serum Sodium Category. Legend. Grouped bar chart displaying median length of stay (LOS) in days stratified by day of hospital admission and serum sodium category among 38,474 admission episodes. Serum sodium categories are defined as: normonatremia (135−145 mmol/L, green bars), mild hyponatremia (130-134.9 mmol/L, yellow bars), moderate hyponatremia (125-129.9 mmol/L, orange bars), and severe hyponatremia (<125 mmol/L, red bars). Error bars represent interquartile ranges (25th-75th percentiles). Admissions with moderate and severe hyponatremia generally exhibit longer median LOS compared to those with normonatremia or mild hyponatremia. This pattern is consistent across all days of the week. S11 Table shows the variation in serum sodium concentration with admission day while S12 Table shows the variation in severity of hyponatremia with admission day.

The general trends were similar between groups. The LOS was lowest for Monday admissions although not significantly different from Sunday admissions’ LOS (S6 and S7 Tables). The LOS generally increased from Mondays’ admissions to Fridays’ followed by a decrease from Saturdays’ admissions to Mondays’ (Fig 4, Tables 2, S6 and S7).

In patients with normonatremia, the LOS of Friday admissions was significantly longer than that of patients admitted on any other day except Thursday (S6 Table). The LOS of Thursday admissions was significantly longer than that of admissions on Sunday, Monday or Tuesday. In patients with hyponatremia, the LOS of Monday admissions was significantly shorter than that of admissions on every other day except Sunday (S7 Table). Similarly, the LOS of Sunday admissions was significantly shorter than that of all days except Monday and Saturday.

The association between admission day with LOS varied with hyponatremia severity. As shown in S8–S10 Tables, moderately hyponatremic patients had longer LOS (mean 8.03 ± SD 5.99 days; median 6, IQR 4–11) than mildly (mean 7.6 ± SD 5.86 days; median 6, IQR 3–10) or severely (7.73 ± SD 5.69; median 6, IQR 4–9) hyponatremic patients (p = 1.5x10^-6^; S7 Table). The effect of admission day on LOS in each cohort was compared using Kruskal-Wallis tests followed by Dunn’s tests. These analyses revealed statistically significant differences in LOS between at least two subgroups of those with mild (H(6)=38.1, p = 9.13x10^-7^) and moderate (H(6)=18.4, p = 0.0051) hyponatremia, but not in severe hyponatremia (H(6)=3.25, p = 0.77).

### Regression analyses

A total of 38,474 patient admission episodes were included in the multivariable regression analyses (Table 4). The comprehensive models investigated factors associated with LOS, ITU admission, and inpatient mortality, adjusting for age, sex, day-of-week on admission, serum sodium concentrations, the COVID-19 pandemic period, and hospital site.

#### ITU admission.

The multivariable logistic regression model (Akaike Information Criterion (AIC) 39,537; McFadden R² 0.043) identified several factors associated with ITU admission (Table 4). The Hosmer-Lemeshow goodness-of-fit test indicated a significant lack of fit (X^2^ [8]=52.77, p < 0.001), suggesting discrepancies between observed and predicted frequencies of ITU admission across risk strata. Adjusted odds ratios (AOR) should be interpreted as directional indicators rather than precise estimates of absolute risk.

Age: Each additional year increased the odds of ITU admission by less than 1% (AOR = 1.00; 95%CI: 1.00–1.00; p = 0.011).

Sex: Female admissions had lower odds of ITU admission compared to males (AOR = 0.87; 95%CI: 0.83–0.92; p < 0.001).

Day of Admission: Significant differences in the odds of ITU admission were observed across the days of the week (p < 0.001). Compared to Sunday (reference), admissions on Thursday (AOR = 1.21; 95%CI: 1.11–1.33), Friday (AOR = 1.17; 95%CI: 1.06–1.28), and Saturday (AOR = 1.33; 95%CI: 1.21–1.46) had significantly higher odds of ITU admission. Monday, Tuesday and Wednesday showed no significant differences compared to Sunday.

Sodium Severity: No statistically significant association with ITU admission (p = 0.062).

COVID-19 Pandemic Period: Admission during the COVID period was not significantly associated with the odds of ITU admission compared to the pre-COVID period (AOR = 0.97; 95%CI: 0.92–1.02; p = 0.20).

Hospital Site: Significant differences in the odds of ITU admission were observed (p < 0.001). Compared to the admissions in Riyadh (reference), the admissions hospital in Medina (AOR = 1.92; 95%CI: 1.78–2.06), Dammam (AOR = 2.18; 95%CI: 1.96–2.41), and Al Ahsa (AOR = 4.20; 95%CI: 3.91–4.51) had significantly higher odds of ITU admission.

#### Inpatient mortality.

A separate multivariable logistic regression model (AIC 12,872; McFadden R² 0.199) was developed to assess factors associated with inpatient mortality (Table 4). The Hosmer-Lemeshow goodness-of-fit test indicated a significant lack of fit (X^2^ [8]=38.12, p < 0.001), highlighting differences between observed and predicted mortality rates. Consequently, AOR should be interpreted as directional indicators rather than precise estimates of absolute risk.

Age: Each additional year, the odds of inpatient mortality increased by approximately 4% (AOR = 1.04; 95%CI: 1.03–1.04; p < 0.001).

Sex: Female admissions had slightly lower odds of inpatient mortality compared to males (AOR = 0.88; 95%CI: 0.80–0.97; p = 0.010).

ITU Admission: ITU admission was strongly associated with inpatient mortality (AOR = 11.90; 95%CI: 10.70–13.20; p < 0.001).

Day of Admission: No statistically significant differences (p = 0.50).

Sodium Severity: Overall, o statistically significant association (p=0.13).

COVID-19 Pandemic Period: Admission during the COVID period was associated with significantly higher mortality than admission during the pre-COVID period (AOR = 1.41; 95%CI: 1.28–1.55; p < 0.001).

Hospital Site: Significant differences in inpatient mortality were observed across hospital sites (p < 0.001). Compared to the admissions in Riyadh (reference), the admissions to hospital in Medina (AOR = 0.78; 95%CI: 0.67–0.90), Damman (AOR = 0.41; 95%CI: 0.30–0.54), and Al Ahsa (AOR = 0.70; 95%CI: 0.60–0.80) had significantly lower inpatient mortality.

#### Length of stay (LOS).

The LOS was modelled using a Gamma GLM with log link (Table 4). This model was selected over a Negative Binomial GLM due to its lower AIC (215,565 vs. 218,564). The model explained 12% of LOS variance (pseudo‑R² = 0.12). The model demonstrated several significant associations with LOS (Table 4).

Age: For each additional year, the expected LOS increased by approximately 1% (Rate Ratio (RR)=1.01; 95%CI: 1.01–1.01; p < 0.001).

Sex: Female admissions had an expected LOS approximately 1% longer than males (RR = 1.01; 95%CI: 0.99–1.02; p = 0.40).

Day of Admission: Significant differences in expected LOS were observed across days of the week (p < 0.001). Compared to Sunday (reference), expected LOS was notably longer for admissions on Tuesday (RR = 1.06; 95%CI: 1.03–1.09), Thursday (RR = 1.08; 95%CI: 1.05–1.11), and Friday (RR = 1.10; 95%CI: 1.07–1.13).

Sodium Severity: Significant complex non-linear association with LOS (p < 0.001).

ITU Admission: Patients admitted to an ITU had an expected LOS approximately 69% longer than those not admitted to an ITU (RR = 1.69; 95%CI: 1.66–1.72; p < 0.001).

Mortality: Admission episodes during which the patients died had an expected LOS approximately 20% longer than those admission episodes in which the patient survived (RR = 1.20; 95%CI: 1.16–1.25; p < 0.001).

COVID-19 Pandemic Period: Admission during the COVID period was associated with a slightly shorter expected LOS compared to the pre-COVID period (RR = 0.98; 95%CI: 0.97–1.00; p = 0.014).

Hospital Site: Significant differences in expected LOS were observed across hospital sites (p < 0.001). The LOS of admissions in Riyadh (reference), was longer than that of admissions tohospital in Medina (RR = 0.90; 95%CI: 0.88–0.93), Dammam (RR = 0.75; 95%CI: 0.72–0.77), and Al Ahsa (RR = 0.84; 95%CI: 0.82–0.86).

Diagnostic plots for the Gamma GLM revealed heteroscedasticity, with the variance of residuals increasing with predicted LOS and the Q-Q plot indicated right-skew in the residuals’ upper tail. These observations suggest imperfect modeling of extreme variability and very long LOS.

#### Multicollinearity assessment.

Variance inflation factors (VIFs) were calculated for all predictors in all three multivariable models (LOS, ITU Admission, and Inpatient Mortality). All generalized VIF (GVIF) values were consistently low (below 1.2 across all models), indicating no significant multicollinearity among the independent variables, ensuring the stability of the coefficient estimates.

The apparent inconsistencies between the descriptive and regression analyses stem from the regression adjustment for age, sex, and ITU admission. Furthermore, regression quantifies the magnitude and direction of the associations of each variable, holding others constant, while the descriptive analyses provide unadjusted trends. This difference in analytical approach can lead to differing interpretations of the significance of observations.

### Sensitivity analysis relating to admission before or during COVID-19 pandemic

The baseline demographics of the study population stratified by admission before or during the COVID-19 period are presented in S14 Table. To assess robustness of primary findings regarding the association of admission day with patient outcomes, we conducted comparative sensitivity analyses incorporating interaction terms between admission day and the defined COVID-19 pandemic period using multivariable regression models, adjusted for age, sex, ITU admission status (for LOS and mortality models), serum sodium concentration, and hospital site (S15 Table). Hypernatremic patients (sodium >145 mmol/L) were excluded from these analyses.

Despite increased inpatient mortality during the COVID-19 pandemic period (1082 (7.2%); pre-COVID 1346 (5.3%); log rank χ^2^ = 54.1 p = 2x10^-13^), formal interaction tests revealed no statistically significant interactions between admission day and the COVID period for ITU admission (global p = 0.542) or LOS (global p = 0.270). The global interaction test for inpatient mortality was marginally significant (p = 0.037).

#### Interaction of admission day with COVID‑19 period.

To evaluate whether the COVID-19 pandemic modified the relationship between admission day and patient outcomes, we examined interaction terms for all three outcome measures (S15 Table).

ITU admission: The global interaction test was not significant (p = 0.542) and all individual interaction terms had 95%CIs crossing one (range: 0.88–1.03), indicating that the association between admission day and ITU admission remained consistent across the pre-COVID and COVID periods.

Inpatient mortality: Despite the marginally significant global interaction test (p = 0.037), the individual interaction terms showed mixed findings. Thursday admissions during COVID, was associated with significantly reduced mortality risk compared to pre-COVID (interaction OR=0.59, 95%CI: 0.42–0.84, p = 0.004), as were Monday admissions (interaction OR=0.69, 95%CI: 0.48–0.98, p = 0.037). However, other days showed no significant interactions. The inconsistent pattern suggests that any modification of the admission day-mortality relationship by the COVID period was modest and inconsistent. The Kaplan-Meier survival curves (S2 Fig) illustrate the survival probabilities of admissions before or during the COVID period.

LOS: The formal interaction test for LOS was not significant (p = 0.270). All individual interaction terms showed no statistically significant modifications after adjustment (95%CI range for IRRs: 0.94–1.11, all crossing or approaching one). This indicates that the association between admission day and LOS remained stable across the pre-COVID and COVID periods. These associations with LOS are shown in S3 Fig.

This sensitivity analysis provides critical insights. The associations of admission day with patient outcomes remained largely consistent across the Pre-COVID and COVID periods. The global interaction tests were not significant for both ITU admission (p = 0.542) and LOS (p = 0.270).

Independent of admission day, admission during the COVID-19 period was associated with increased mortality risk. In the primary model (Table 4), this represented a 41% increase in odds (OR=1.41, 95%CI: 1.28–1.55). In sensitivity analyses including day×COVID interactions (S15 Table), the main effect coefficient (representing Sunday admissions) was OR=1.74 (95%CI: 1.37–2.21, p < 0.001), with other weekdays showing variable effects through interaction terms (Monday OR=0.69, Thursday OR=0.59). Thus, the increased mortality observed during the COVID period was an additive main effect rather than a differential impact by admission day, supporting the generalizability of the primary findings.

## Discussion

This multicentre cohort study of 40,366 medical admissions examined associations between admission day, hyponatremia, markers of illness severity (ITU admission and severity of hyponatremia), and patient outcomes (LOS, mortality). While some nuances emerged, the overall temporal trends were similar between groups. There are three key findings: (i) hyponatremia occurred in 42% of medical admissions, (ii) Friday admissions associated with the longest LOS irrespective of sodium status, and (iii) the association between admission day and LOS attenuated as hyponatremia severity increased, suggesting prioritization of critically ill patients.

### Length of stay

Friday admissions had the longest LOS, independent of serum sodium concentration. Monday admissions had the shortest LOS. The LOS generally increased from Monday to Friday, then decreased from Saturday to Sunday. This pattern, where Sunday is the first working day in Saudi Arabia, may reflect the need to address weekend backlogs, explaining why Sunday admissions’ LOS was not shorter than Mondays’.

Tuesday admissions’ LOS was unexpectedly longer than expected. This could be linked to our institutions’ internal medicine residency training program, with teaching activities held on Tuesday afternoons, reducing physician availability.

Besides Friday admission episodes, hyponatremic admissions had longer LOS than normonatremic admissions (p < 0.0001), consistent with previous studies [11–14]. This likely reflects established guidelines that limit the rate of correction of hyponatremia to reduce the risk of osmotic demyelination syndrome [1,2,15]. However, if guidelines were the only factor, LOS should increase with worsening hyponatremia. Yet, the association between LOS and admission day was attenuated as the severity of hyponatremia increased (S6–S9 Tables), suggesting prioritization of sicker patients. The shorter LOS in severe hyponatremia may also reflect higher early mortality (Table 3).

The findings of a recent retrospective study were interpreted to suggest that the recommendations restricting the rate of correction of hyponatremia may be overcautious [16]. Relaxing these guidelines may reduce the impact of hyponatremia on LOS. However, many experts argued against changing the existing guidelines, highlighting analytical flaws and citing concerns about the risk of osmotic demyelination syndrome with rapid correction of chronic hyponatremia [17].

### Admission day, severity of hyponatremia, ITU admission and mortality

Mean serum sodium concentrations varied slightly with admission day, but differences were not clinically significant (Table 2). However, a greater proportion of admissions on Thursdays or Fridays had severe hyponatremia compared to Sunday-Wednesday (S5 Table).

### Mortality findings and limitations

Mortality showed no significant association with admission day or serum sodium concentration in adjusted analyses (Table 4). However, several methodological limitations warrant consideration. With 5% mortality across 40,354 admissions, post-hoc power calculations indicate only 58% power to detect 10% relative risk reductions (α = 0.05). Clinically relevant smaller differences may exist but remain undetected.

The absence of validated comorbidity indices may confound these associations. Additionally, our hospitals provide 24-hour senior physician coverage, potentially blunting weekend-related mortality effects observed in systems with reduced weekend staffing. The absence of mortality signals should be viewed as study limitations rather than evidence that admission day or hyponatremia do not affect mortality.

### ITU admission patterns

The ITU admission rates generally increased from Sunday to Saturday admissions (Table 2). Notably, Friday admissions with hyponatremia showed significantly lower ITU admission rates than normonatremic Friday admissions (p = 0.005), warranting further investigation into potential differences in triage patterns or patient flow at weekends.

ITU admission rates varied modestly by hyponatremia severity (mild 23.6%, moderate 21.9%, severe 22.0%; p = 0.043; Table 3), though absolute differences were small. This pattern may reflect competing factors: while severe hyponatremia indicates greater physiological derangement, patients with mild hyponatremia may present with complex comorbidities requiring intensive monitoring. Conversely, severely hyponatremic patients may be deemed too frail for aggressive intervention or have advanced directives limiting treatment escalation. Hyponatremia appears to function as a marker of systemic illness rather than the primary driver of ITU admission decisions.

The need for ITU admission is a complex variable. This subjective marker of illness severity may be considered an outcome measure, but bed availability may influence triage and goals-of-care decisions potentially confounding findings [18–20]. Thus, our observations may reflect temporal variations in ITU bed capacity and clinicians’ perceptions of long-term outcomes associated with severity of hyponatremia [11–13].

### Weekend effect vs. day-to-day variations

While the “weekend effect” is well-recognized [6–9], our data reveal that outcomes vary across all of the days of the week, not just weekends. Our findings suggest that institutional scheduling and staffing patterns create day-specific variations that extend beyond the conventional weekend-weekday dichotomy. Residency training schedules exemplify this, as analyses that solely compared weekends to weekdays may overlook such complexities.

### Association of COVID-19 pandemic with worse outcomes: Sensitivity analysis implications

The sensitivity analysis exploring admission day associations during the defined COVID-19 pandemic period provided critical insights. Admission day associations with inpatient mortality and ITU admission remained largely consistent across the pre-COVID-19 and COVID-19 periods.

The COVID-19 sensitivity analysis revealed a significant global interaction term for mortality (p = 0.037), but only Thursday and Monday admissions showed individual significant interactions (both indicating reduced mortality during COVID: Thursday OR=0.59, p = 0.004; Monday OR=0.69, p = 0.037). Other weekdays showed no significant interactions. Systemic pandemic-related changes in care delivery, case-mix, or resource allocation may have subtly influenced admission-day mortality associations without producing statistically robust day-specific effects. Larger prospective studies with comprehensive comorbidity measures and standardised severity-of-illness scores are required to definitively evaluate these relationships. The ITU admission global interaction term was not significant (p = 0.542), confirming stable associations across both periods.

The LOS global interaction test was not significant (p = 0.270). The 95%CIs intervals of all individual interaction terms include one (IRR range: 1.00–1.05; 95%CI: 0.94–1.11). This definitively demonstrates that admission day-LOS associations remained stable throughout the pandemic period, despite the primary model showing an overall 2% reduction in LOS during the COVID-19 pandemic period (IRR = 0.98, p = 0.014).

The main impact of the COVID-19 pandemic manifested as additive main effects rather than differential day-specific patterns. Mortality increased substantially (OR=1.74, 95%CI: 1.37–2.21, p < 0.001). The LOS decreased slightly (IRR = 0.98, 95%CI: 0.97–1.00, p = 0.014). Rates of ITU admission remained unchanged (OR=1.00, p = 0.971). Thus, systemic pandemic-related changes in care delivery, case-mix, or resource allocation occurred uniformly across weekdays rather than disproportionately affecting specific admission days.

### Patient‐perceived stress and the “weekend effect”

The timing of hospital admission can influence patients’ psychological wellbeing. Patients admitted when staffing is reduced report lower satisfaction with communication potentially because of perceived delays in evaluation and treatment [21]. Heightened psychological stress in hospitalized patients is associated with worse outcomes [22]. In patients with low back pain admitted on Fridays or Saturdays, greater distress correlated with prolonged LOS [23]. In our cohort, Friday and Saturday admissions coincided with the statutory weekend in Saudi Arabia, when senior‑physician coverage and ancillary services are scaled back. Although our data do not include validated stress‑or anxiety scores, the consistently longer LOS for weekend admissions suggest that patients may have experienced additional uncertainty and distress while awaiting investigations or therapeutic decisions.

Recognising this psychosocial dimension is important. Heightened stress can impair recovery, increase pain perception, and hinder discharge planning. Future prospective work should incorporate patient‑reported outcome measures (e.g., Hospital Anxiety and Depression Scale, visual‑analogue stress scales) to quantify the emotional impact of weekend admissions and guide interventions such as enhanced bedside communication, early‑warning rounding, or targeted support services on high‑risk days.

### Implications for clinical practice, unanswered questions and future research

Understanding day-to-day variations can inform strategies to optimize patient outcomes and resource allocation. Scheduling resident activities to minimize disruptions and standardizing care delivery across weekdays and weekends, could be beneficial. For example, targeted augmentation of staffing on Fridays and streamlined discharge processes on Tuesdays could reduce LOS by up to 0.5 days per admission, translating into substantial bed‑capacity gains.

These findings have informed institutional initiatives aimed at mitigating the “weekend effect” on patient outcomes. While the broader phenomenon is recognized by key stakeholders, including the Ministry of Health and the Ministry of National Guard – Health Affairs, the specific results of our analysis have supported a series of targeted interventions at our institution.

The Internal Medicine Section and the Residency Program leadership implemented several evidence-based changes to improve the quality and continuity of care on weekends:

- The appointment of Hospital Medicine Fellows provides essential senior-level oversight, complementing resident teams and enhancing patient management during periods of reduced staffing.
- Daily 5 PM consultant ward rounds ensure the timely review of new admissions, facilitating the early identification and management of high-risk patients.
- The introduction of twice-daily post-discharge follow-up clinics has strengthened care transitions and reduced readmission risk, a particular concern for weekend discharges.

These proactive measures illustrate institutional commitment to patient safety and quality improvement. They are powerful examples of translating data-driven research into meaningful clinical action, directly addressing the systemic challenges associated with the “weekend effect.” Future studies should explore interventions to standardize the quality of care and investigate the combined impact of admission day and residency schedules on patient outcomes.

### Strengths and weaknesses of the study

To our knowledge, this is the ﬁrst study exploring how speciﬁc admission days influence outcomes in hyponatremic patients. However, the retrospective observational design precludes causal inference. While we identified robust associations, the lack of detailed diagnostic data, comprehensive comorbidity measures, and information on the precise duration of hyponatremia (acute vs. chronic) at presentation limits definitive conclusions. These unmeasured confounders may influence both admission patterns and outcomes. Our statistical models, although adjusted for available variables, cannot fully mitigate such biases.

#### Model calibration and goodness-of-fit.

The Hosmer-Lemeshow goodness-of-fit tests indicated significant lack of fit (p < 0.001) for all logistic regression models (ITU admission and inpatient mortality), suggesting potential calibration issues, over-dispersion, or unmeasured confounding. The observed and predicted probabilities were not fully aligned, likely reflecting incomplete model specification. Although we adjusted for key demographic and clinical variables, residual confounding by unmeasured factors (comorbidity burden, functional status, code status preferences) cannot be excluded. We therefore interpret the reported odds ratios as indicating direction of association rather than precise absolute risk estimates. Future studies incorporating validated comorbidity indices (e.g., Charlson Comorbidity Index, Elixhauser score) and penalised regression techniques would strengthen model robustness.

#### Length-of-stay model limitations.

The Gamma GLM of LOS also showed significant lack of fit, particularly in the upper tail of the distribution. Diagnostic plots revealed heteroscedasticity and right-skew in the residuals’ upper tail, indicating suboptimal performance with the prediction of very long hospitalizations. Heteroscedasticity may introduce inefficiency in coefficient estimates and affect standard error precision, potentially inflating type I error rates for extreme LOS values. While robust standard errors partially mitigate this concern, the residual right-skew suggests that extreme LOS were not fully captured. Unmeasured factors (e.g., social barriers to discharge, rare complications, or institutional care processes) may have contributed to prolonged admissions.

The pseudo-R² value of 0.12 indicates that admission day, sodium concentration, and measured covariates explain only 12% of LOS variance, underscoring substantial influence from unmeasured factors (disease-specific characteristics, social determinants, discharge-planning efficiency). Future analyses might benefit from alternative modeling strategies (zero-truncated negative binomial regression, quantile regression, or log-normal distributions) to better accommodate skewness and variance heterogeneity, or multilevel models accounting for within-patient clustering of repeated admissions.

#### Statistical power.

With 2,428 deaths among 40,366 admissions (6% mortality), the study had 58% power (α = 0.05) to detect a 10% relative reduction in mortality (OR≈0.9). This limits our ability to detect clinically meaningful mortality differences and explains why we observed no significant association between admission day or sodium concentration and mortality despite numerical trends. The relatively low event rate restricts the precision of mortality estimates. Larger multicentre prospective studies are needed to definitively evaluate mortality associations.

#### Interaction terms.

Interpretation of the interaction between admission day and COVID-19 period requires caution. For LOS, the non-significant global test (p = 0.270) coupled with non-significant individual interactions definitively demonstrates stable associations across pandemic periods. The marginally significant global interaction test for mortality (p = 0.037) contrasted with mostly non-significant individual day-specific contrasts, although Thursday and Monday admissions achieved significance. While the overall pattern suggests some differential COVID-19 impact for mortality, individual coefficient confidence intervals were wide, and replication in independent cohorts is warranted.

#### Generalisability.

Our findings derive from a nationally funded health system in Saudi Arabia with standardised staffing policies across sites. Extrapolation to privately funded hospitals, systems with different weekend structures (e.g., Saturday-Sunday weekends), or healthcare settings with variable senior physician coverage should be undertaken cautiously. The statutory Friday-Saturday weekend in Saudi Arabia creates distinct temporal patterns of staffing and resource availability compared with Western healthcare systems.

## Conclusion

This multicentre cohort demonstrates that admission day and hyponatremia independently associate with hospital LOS. Friday admissions have the longest stays independent of serum sodium status. The association between admission day and LOS attenuates as hyponatremia severity increases, suggesting prioritisation of critically ill patients mitigates some weekend-effect impact. Unexpectedly prolonged Tuesday LOS may reflect institutional factors (resident teaching activities) highlighting that ‘weekend effects’ represent broader temporal workflow variations rather than simple weekend/weekday dichotomies.

Mortality showed no significant association with admission day or sodium levels in adjusted analyses, though the study was underpowered for this outcome. The COVID-19 pandemic did not fundamentally alter admission-day associations with mortality, ITU admission or LOS. The increased mortality represented an additive main effect rather than a differential day-specific impact.

These findings support targeted interventions: enhanced mid-week staffing during resident teaching periods, standardised weekend care protocols, and early identification systems for patients at risk of prolonged LOS. Future prospective studies with detailed severity-of-illness measures (comorbidity indices, APACHE scores) are needed to establish causality and guide evidence-based workforce allocation strategies.

**Key messages**

**What is already known on this topic:** Hyponatremia is common in hospitalized patients and is associated with worse outcomes. The weekend effect shows higher mortality and altered LOS for weekend admissions. This study was needed to explore how admission day and sodium levels jointly affect outcomes in a setting where the weekend is Friday-Saturday.

**What this study adds:** This study reveals that hyponatremia and admission day are independently and jointly associated with LOS and need for ITU admission, but not mortality. The classic “weekend effect” is part of a broader temporal pattern. Friday admissions have the longest LOS regardless of sodium levels. However, unexpected prolongation of Tuesdays’ admissions’ LOS indicates the potential influence of institutional workflows (e.g., residents’ teaching activities).

**How this study might affect research, practice, or policy:** Insights from this study can guide targeted interventions based on admission day and sodium levels, improving patient management and resource utilization, and addressing the effect of admission day.

**Key points**

Admission day (not just weekends) affects outcomes. LOS varies, with Fridays highest and Mondays lowest. Resident teaching may explain the longer LOS of Tuesday admissions.

Hyponatremia is associated with incrementally prolonged LOS (except Fridays' admissions). However, this effect is less pronounced in patients with severe hyponatremia, suggesting prioritization of sicker patients.

ITU admission rates increase from Sundays to Saturdays. Hyponatremic patients admitted on Fridays had lower ITU admission rates than normonatremic Friday admissions (OR 0.82, p = 0.005 ITU). Mild hyponatremia is linked to higher ITU admission rates.

The classic “weekend effect” is part of a broader temporal pattern. Tuesdays’ unexpected prolongation of LOS highlights the influence of institutional workflow (e.g., resident teaching). Understanding day-to-day variations can guide strategies to improve patient care and resource allocation.

Scheduling resident activities to minimize disruptions and standardizing care delivery across all weekdays may be beneficial.

## Supporting information

S1 Table Intensive therapy unit (ITU) admissions stratified by admission day, serum sodium and hospital. Legend. This table compares the number of admissions with hyponatremia (serum sodium <135 mmol/l) upon admission transferred to an intensive therapy unit (ITU) with that of admissions who were normonatremic (135–145 mmol/L). Data are stratified by the day of admission and hospital site. Data are presented as frequency and percentage with 95% confidence intervals (CI).
(PDF)

S2 Table Inpatient mortality stratified by admission day, serum sodium and hospital. Legend. This table compares the inpatient mortality of admissions with hyponatremia (serum sodium <135 mmol/l) with that of admissions who were normonatremic (135–145 mmol/L). Data are stratified by the day of admission and hospital site. Data are presented as frequency and percentage with 95% confidence intervals (CI).
(PDF)

S3 Table Length of stay stratified by admission day and hospital. Legend. This table compares the length of stay of admissions with hyponatremia (serum sodium <135 mmol/l) upon admission with that of admissions who were normonatremic (135–145 mmol/L). Data are stratified by day of admission and hospital site. Data are presented as mean ± standard deviation (SD) and median (interquartile range; IQR).
(PDF)

S4 Table The associations of admission day with ITU admissions in normonatremic patients. Legend. A chi-squared test demonstrated statistically significant association between the day of admission and the numbers of normonatremic medical admissions transferred to an intensive therapy unit (ITU; p < 0.00000001). This finding indicates that the distribution of ITU admissions varies significantly with the day of admission, rather than establishing a causal link. In Saudi Arabia the weekend is Friday-Saturday, while Sunday to Thursday are weekdays. Serial post hoc testing with pairwise Chi-squared tests is shown. Statistically significant differences (p < 0.05) are indicated (*).
(PDF)

S5 Table The associations of admission day with ITU admissions in hyponatremic patients. Legend. A chi-squared test demonstrated statistically significant association between the day of admission and the numbers of hyponatremic medical admissions transferred to an intensive therapy unit (ITU; p = 0.0036). This finding indicates that the distribution of ITU admissions in this cohort varied significantly with the day of admission, rather than establishing a causal link. In Saudi Arabia the weekend is Friday-Saturday, while Sunday to Thursday are weekdays. Serial post hoc testing with pairwise Chi-squared test is shown. Statistically significant differences (p < 0.05) are indicated (*).
(PDF)

S6 Table Association between length of stay of normonatremic patients and admission day. Legend. This table presents the association between day of admission and the length of stay (LOS) of admissions with normonatremia. The LOS is presented as Mean± standard deviation (SD) and Median (interquartile range (IQR)). The Kruskal-Wallis test demonstrated significant association of admission day with the LOS of normonatremic medical inpatients (p = 1.28E-31), indicating that LOS varies significantly based on the day of admission. This observation does not imply a causal relationship. Post hoc testing with Dunn’s test is shown. Statistically significant differences are indicated (*).
(PDF)

S7 Table Association between length of stay of hyponatremic patients and admission day. Legend. This table shows the association between admission day and the length of stay (LOS) of admissions with hyponatremia. The LOS is presented as mean±standard deviation (SD) and median (interquartile range (IQR)). The Kruskal-Wallis test demonstrated a statistically significant association between day of admission and the LOS of hyponatremic medical admissions (p = 5.48x10^-9^). This observation does not imply a causal relationship. Post hoc testing with Dunn’s test is shown. Statistically significant differences are indicated (*).
(PDF)

S8 Table Association between length of stay and admission day in mildly hyponatremic patients. Legend. This table shows the association between admission day and the length of stay (LOS) of admissions with mild hyponatremia. The LOS is presented as Mean± standard deviation (SD) and Median (interquartile range (IQR)). The Kruskal-Wallis test demonstrated statistically significant association between admission day and the LOS of mildly hyponatremic medical inpatients (serum sodium 130–134.9 mmol/L; p = 5.48x10^-9^). Post hoc testing with Dunn’s test is shown. These tests do not imply a causal relationship. Statistically significant differences are indicated (*).
(PDF)

S9 Table Association between length of stay and admission day in moderately hyponatremic patients. Legend. This table shows the association between admission day and the length of stay (LOS) of admissions with moderate hyponatremia. The LOS is presented as Mean± standard deviation (SD) and Median (interquartile range (IQR)). The Kruskal-Wallis test demonstrated a statistically significant association between the day of admission and the LOS of moderately hyponatremic medical admissions (serum sodium 125–129.9 mmol/L; p = 5.48x10^-9^). Post hoc testing with Dunn’s test is shown. Statistically significant differences are indicated (*).
(PDF)

S10 Table Association between length of stay and admission day in severely hyponatremic patients. Legend. This table shows the association between admission day and the length of stay (LOS) of admissions with severe hyponatremia. The LOS is presented as mean± standard deviation (SD) and median (interquartile range (IQR)). The Kruskal-Wallis test demonstrated a statistically significant association between the day of admission and the LOS of severely hyponatremic medical admissions (serum sodium < 125 mmol/L; (p = 0.88). Post hoc testing with Dunn’s test is shown. There were no statistically significant differences (p < 0.05).
(PDF)

S11 Table The variation in serum sodium concentration with day of admission. Legend. This table shows the variation in serum sodium concentration with admission day. In Saudi Arabia the weekend is Friday-Saturday, while Sunday to Thursday are weekdays. Data presented as mean ± standard deviation (SD) and median and interquartile range (IQR). The differences were compared using the Kruskal-Wallis test. This revealed statistically significant differences in distribution of serum sodium between at least two days (H(6)=54.9, p = 4.41x10^-10^). Serial post hoc testing with Dunn’s test is shown. Statistically significant differences are indicated (*).
(PDF)

S12 Table The variation in severity of hyponatremia with day of admission. Legend. This table shows the variation in severity of hyponatremia with admission day. In Saudi Arabia the weekend is Friday-Saturday, while Sunday to Thursday are weekdays. The differences were compared using Chi-squared tests. This revealed statistically significant differences in the severity of hyponatremia between at least two days. Serial post hoc testing with pairwise chi-squared tests is shown. Statistically significant differences after the application of Bonferroni correction (p < 0.0071; i.e., 0.05/7) are indicated (*).
(PDF)

S13 Table The patients discharged before the next weekend stratified by day of admission and serum sodium. Legend. This table shows the variation in the number of admissions before the next weekend stratified by serum sodium concentration and admission day. In Saudi Arabia the weekend is Friday-Saturday, while Sunday to Thursday are weekdays. The differences were compared using Chi-squared tests. This revealed statistically significant differences in the percentages of admission episodes discharged before the next weekend between at least two days. Serial post hoc testing with pairwise chi-squared tests is shown. Statistically significant differences after the application of Bonferroni correction (p < 0.0071; i.e., 0.05/7) are indicated (*).
(PDF)

S14 Table Demographics, serum sodium on admission and outcomes of the whole study population stratified by admission before or during COVID-19 pandemic period. Legend. This table presents the demographics, serum sodium, intensive therapy unit (ITU) admissions and outcomes (length of stay, and mortality) of the study population stratified by admission before (pre-COVID-19) or during the COVID-19 period. Data are presented as mean ± standard deviation (SD) or frequency and percentages (%) as appropriate.
(PDF)

S15 Table Multivariable Regression Models for Inpatient Mortality, ITU Admission, and Length of Stay (LOS): Sensitivity Analysis for COVID Period vs. Pre-COVID. Legend. Results of multivariable regression analysis examining the association between age, sex, admission day, serum sodium and admission to the intensive therapy unit (ITU), mortality or length of stay (LOS). Three models were constructed: logistic regression for intensive therapy unit (ITU) admission and inpatient mortality (presented as adjusted odds ratios (OR) with 95% Confidence Intervals (CI)) and gamma regression for LOS (presented as incidence rate ratios (IRR) with 95%CI). These analyses serve as a sensitivity analysis to assess the impact of the COVID-19 period on these outcomes and how it interacts with other predictors. Dummy coding (one-hot encoding) was used for day-of-week variables using Sunday as the reference. In Saudi Arabia the weekend is Friday-Saturday, while Sunday to Thursday are weekdays. Male was the reference for sex. The pre-COVID period was the reference for the COVID period. P-values are based on two-tailed tests. Note: Model fit statistics differ from Table 4 due to inclusion of interaction terms. Model fit statistics: Mortality model (N = 38,474; AIC = 12,863; BIC = 13,042). ITU Admission model (N = 38,474; AIC = 39,545; BIC = 39,716). LOS model (N = 38,474; AIC = 215,775; BIC = 215,963). Abbreviations: AIC, Akaike Information Criterion; BIC, Bayesian Information Criterion; N, Number of Observations.
(PDF)

S1 File STROBE checklist. This file contains the completed Strengthening the Reporting of Observational Studies in Epidemiology (STROBE) checklist for the final submitted manuscript. In accordance with the STROBE guidelines for reporting observational studies, each item is addressed with reference to the corresponding section or table in the main text.
(PDF)

S1 Fig The patients discharged before the next weekend stratified by day of admission and serum sodium. Legend. This figure shows the variation in the percentage of admission episodes discharged before the next weekend stratified by serum sodium concentration and admission day. In Saudi Arabia the weekend is Friday-Saturday, while Sunday to Thursday are weekdays. The differences were compared using Chi-squared tests. This revealed statistically significant differences in the percentages of admission episodes discharged before the next weekend between at least two days. Serial post hoc testing with pairwise chi-squared tests is shown. Statistically significant differences after the application of Bonferroni correction (p < 0.0071; i.e., 0.05/7) are indicated (*).
(TIF)

S2 Fig Kaplan-Meier survival analysis comparing patients admitted before or during the COVID-19 period. Legend. This Kaplan-Meier analysis assessed the 30-day inpatient survival of admissions before (01/01/16 to 29/02/20 (pre-COVID)) or during the COVID-19 period (01/03/20 to 31/05/22). A statistically significant increase in inpatient mortality during the COVID-19 pandemic period was observed (1082 (7.2%); pre-COVID 1346 (5.3%); log rank χ2 = 54.1 p = 2x10^-13^). No statistically significant differences were observed between groups (log-rank test, p = 0.15). 95% Confidence intervals are shown as shaded areas.
(TIF)

S3 Fig Adjusted mean length of stay stratified by Admission Day and COVID period. Legend. This box plot shows visually compares the length of hospital stay (in days, on a log10 scale) across different admission days, distinguishing between Pre-COVID (blue) and During-COVID (red) periods. Admissions with hypernatremia (serum sodium levels greater than 145 mmol/L) have been excluded from this analysis. Each box indicates the median LOS (horizontal line), the interquartile range (IQR) from the 25th to 75th percentile, and whiskers extending to data within 1.5 times the IQR (outliers are not displayed). The mean LOS is marked within each box (⋆).
(TIF)
