# Supplementary material for: Associations between day of admission, admission hyponatremia and hospital outcomes in medical patients: A retrospective multicenter cohort study
Source: PLoS One. 2025 Oct 27;20(10):e0335248. doi: 10.1371/journal.pone.0335248 (PMC12558553; doi:10.1371/journal.pone.0335248)
Supplement: S1 Table — Legend. This table compares the number of admissions with hyponatremia (serum sodium <135 mmol/l) upon admission transferred to an intensive therapy unit (ITU) with that of admissions who were normonatremic (135–145 mmol/L). Data are stratified by the day of admission and hospital site. Data are presented as frequency and percentage with 95% confidence intervals (CI). (PDF) [file pone.0335248.s001.pdf]

Appendix Table S1. Intensive therapy unit (ITU) admissions stratified by admission day, serum sodium and hospital

Hospital	Riyadh				Medina				Dammam				Al Ahsa			
Sodium	<135 mmol/L		135-145 mmol/L		<135 mmol/L		135-145 mmol/L		<135 mmol/L		135-145 mmol/L		<135 mmol/L		135-145 mmol/L	
Day	N	ITU (N; % (CI)	N	ITU (N; % (CI)	N	ITU (N; % (CI)	N	ITU (N; % (CI)	N	ITU (N; % (CI)	N	ITU (N; % (CI)	N	ITU (N; % (CI)	N	ITU (N; % (CI)
Sun	1901	287; 15.1% (13.5% to 16.7%)	2439	422; 17.3% (15.8% to 18.8%)	329	108; 32.8% (27.8% to 37.9%)	291	65; 22.3% (17.6% to 27.1%)	85	24; 28.2% (18.7% to 37.8%)	231	57; 24.7% (19.1% to 30.2%)	299	157; 52.5% (46.8% to 58.2%)	332	145; 43.7% (38.3% to 49%)
Mon	1906	312; 16.4% (14.7% to 18%)	2489	400; 16.1% (14.6% to 17.5%)	291	89; 30.6% (25.3% to 35.9%)	381	104; 27.3% (22.8% to 31.8%)	79	28; 35.4% (24.9% to 46%)	176	46; 26.1% (19.6% to 32.6%)	257	135; 52.5% (46.4% to 58.6%)	337	143; 42.4% (37.2% to 47.7%)
Tue	1788	301; 16.8% (15.1% to 18.6%)	2476	441; 17.8% (16.3% to 19.3%)	264	74; 28% (22.6% to 33.4%)	374	104; 27.8% (23.3% to 32.3%)	81	25; 30.9% (20.8% to 40.9%)	178	47; 26.4% (19.9% to 32.9%)	277	155; 56% (50.1% to 61.8%)	309	115; 37.2% (31.8% to 42.6%)
Wed	1924	322; 16.7% (15.1% to 18.4%)	2454	481; 19.6% (18% to 21.2%)	230	65; 28.3% (22.4% to 34.1%)	311	67; 21.5% (17% to 26.1%)	97	34; 35.1% (25.6% to 44.5%)	170	37; 21.8% (15.6% to 28%)	234	113; 48.3% (41.9% to 54.7%)	328	136; 41.5% (36.1% to 46.8%)
Thu	1814	342; 18.9% (17.1% to 20.7%)	2191	436; 19.9% (18.2% to 21.6%)	251	76; 30.3% (24.6% to 36%)	293	90; 30.7% (25.4% to 36%)	102	39; 38.2% (28.8% to 47.7%)	188	72; 38.3% (31.3% to 45.2%)	247	130; 52.6% (46.4% to 58.9%)	240	101; 42.1% (35.8% to 48.3%)
Fri	1707	248; 14.5% (12.9% to 16.2%)	1948	401; 20.6% (18.8% to 22.4%)	260	87; 33.5% (27.7% to 39.2%)	261	105; 40.2% (34.3% to 46.2%)	75	35; 46.7% (35.4% to 58%)	124	42; 33.9% (25.5% to 42.2%)	231	122; 52.8% (46.4% to 59.3%)	216	92; 42.6% (36% to 49.2%)

Sat	1790	339; 18.9% (17.1% to 20.8%)	1913	439; 22.9% (21.1% to 24.8%)	271	97; 35.8% (30.1% to 41.5%)	294	78; 26.5% (21.5% to 31.6%)	91	45; 49.5% (39.2% to 59.7%)	171	60; 35.1% (27.9% to 42.2%)	244	133; 54.5% (48.3% to 60.8%)	234	114; 48.7% (42.3% to 55.1%)
Total	12830	2151; 16.8% (16.1% to 17.4%)	15910	3020; 19% (18.4% to 19.6%)	1896	596; 31.4% (29.3% to 33.5%)	2205	613; 27.8% (25.9% to 29.7%)	610	230; 37.7% (33.9% to 41.6%)	1238	361; 29.2% (26.6% to 31.7%)	1789	945; 52.8% (50.5% to 55.1%)	1996	846; 42.4% (40.2% to 44.6%)

Legend to Table S1. This table compares the number of admissions with hyponatremia (serum sodium <135 mmol/l) transferred to an intensive therapy unit (ITU) with that of admissions who were normonatremic (135-145 mmol/L). Data are stratified by the day of admission and hospital site. Data are presented as frequency and percentage with 95% confidence intervals (CI).
