# Supplementary material for: Associations between day of admission, admission hyponatremia and hospital outcomes in medical patients: A retrospective multicenter cohort study
Source: PLoS One. 2025 Oct 27;20(10):e0335248. doi: 10.1371/journal.pone.0335248 (PMC12558553; doi:10.1371/journal.pone.0335248)
Supplement: S2 Table — Legend. This table compares the inpatient mortality of admissions with hyponatremia (serum sodium <135 mmol/l) with that of admissions who were normonatremic (135–145 mmol/L). Data are stratified by the day of admission and hospital site. Data are presented as frequency and percentage with 95% confidence intervals (CI). (PDF) [file pone.0335248.s002.pdf]

Appendix Table S2. Inpatient mortality stratified by admission day, serum sodium and hospital

Hospital	Riyadh				Medina				Dammam				Al Ahsa			
**Sodium**	**<135 mmol/L**		**135-145 mmol/L**		**<135 mmol/L**		**135-145 mmol/L**		**<135 mmol/L**		**135-145 mmol/L**		**<135 mmol/L**		**135-145 mmol/L**	
**Day**	**N**	**Deaths (N; % (CI)**	**N**	**Deaths (N; % (CI)**	**N**	**Deaths (N; % (CI)**	**N**	**Deaths (N; % (CI)**	**N**	**Deaths (N; % (CI)**	**N**	**Deaths (N; % (CI)**	**N**	**Deaths (N; % (CI)**	**N**	**Deaths (N; % (CI)**
Sun	1901	97; 5.1% (4.1% to 6.1%)	2439	124; 5.1% (4.2% to 6%)	329	22; 6.7% (4% to 9.4%)	291	13; 4.5% (2.1% to 6.8%)	85	4; 4.7% (0.2% to 9.2%)	231	9; 3.9% (1.4% to 6.4%)	299	24; 8% (4.9% to 11.1%)	332	28; 8.4% (5.4% to 11.4%)
Mon	1906	96; 5% (4.1% to 6%)	2489	116; 4.7% (3.8% to 5.5%)	291	11; 3.8% (1.6% to 6%)	381	24; 6.3% (3.9% to 8.7%)	79	2; 2.5% (-0.9% to 6%)	176	3; 1.7% (-0.2% to 3.6%)	257	17; 6.6% (3.6% to 9.7%)	337	20; 5.9% (3.4% to 8.5%)
Tue	1788	94; 5.3% (4.2% to 6.3%)	2476	113; 4.6% (3.7% to 5.4%)	264	10; 3.8% (1.5% to 6.1%)	374	19; 5.1% (2.9% to 7.3%)	81	3; 3.7% (-0.4% to 7.8%)	178	5; 2.8% (0.4% to 5.2%)	277	19; 6.9% (3.9% to 9.8%)	309	18; 5.8% (3.2% to 8.4%)
Wed	1924	86; 4.5% (3.5% to 5.4%)	2454	131; 5.3% (4.4% to 6.2%)	230	18; 7.8% (4.4% to 11.3%)	311	23; 7.4% (4.5% to 10.3%)	97	3; 3.1% (-0.4% to 6.5%)	170	3; 1.8% (-0.2% to 3.7%)	234	24; 10.3% (6.4% to 14.1%)	328	14; 4.3% (2.1% to 6.5%)
Thu	1814	114; 6.3% (5.2% to 7.4%)	2191	106; 4.8% (3.9% to 5.7%)	251	13; 5.2% (2.4% to 7.9%)	293	19; 6.5% (3.7% to 9.3%)	102	3; 2.9% (-0.3% to 6.2%)	188	7; 3.7% (1% to 6.4%)	247	13; 5.3% (2.5% to 8%)	240	16; 6.7% (3.5% to 9.8%)
Fri	1707	84; 4.9% (3.9% to 5.9%)	1948	107; 5.5% (4.5% to 6.5%)	260	19; 7.3% (4.1% to 10.5%)	261	16; 6.1% (3.2% to 9%)	75	4; 5.3% (0.2% to 10.4%)	124	1; 0.8% (-0.8% to 2.4%)	231	16; 6.9% (3.7% to 10.2%)	216	13; 6% (2.8% to 9.2%)
Sat	1790	110; 6.1% (5% to 7.3%)	1913	110; 5.8% (4.7% to 6.8%)	271	21; 7.7% (4.6% to 10.9%)	294	19; 6.5% (3.7% to 9.3%)	91	6; 6.6% (1.5% to 11.7%)	171	1; 0.6% (-0.6% to 1.7%)	244	21; 8.6% (5.1% to 12.1%)	234	22; 9.4% (5.7% to 13.1%)
Total	12830	681;5.3% (4.9% to 5.7%)	15910	807; 5.1% (4.7% to 5.4%)	1896	114; 6% (4.9% to 7.1%)	2205	133; 6% (5% to 7%)	610	25; 4.1% (6.8% to 14.9%)	1238	29; 2.3% (1.5% to 3.2%)	1789	134; 7.5% (6.3% to 8.7%)	1996	131; 6.6% (5.5% to 7.6%)

Legend to Table S2. This table compares the inpatient mortality of admissions with hyponatremia (serum sodium <135 mmol/l) with that of admissions who were normonatremic (135-145 mmol/L). Data are stratified by the day of admission and hospital site. Data are presented as frequency and percentage with 95% confidence intervals (CI).
