# Supplementary material for: Associations between day of admission, admission hyponatremia and hospital outcomes in medical patients: A retrospective multicenter cohort study
Source: PLoS One. 2025 Oct 27;20(10):e0335248. doi: 10.1371/journal.pone.0335248 (PMC12558553; doi:10.1371/journal.pone.0335248)
Supplement: S3 Table — Legend. This table compares the length of stay of admissions with hyponatremia (serum sodium <135 mmol/l) upon admission with that of admissions who were normonatremic (135–145 mmol/L). Data are stratified by day of admission and hospital site. Data are presented as mean ± standard deviation (SD) and median (interquartile range; IQR). (PDF) [file pone.0335248.s003.pdf]

Appendix Table S3. Length of stay stratified by admission day and hospital

Hospital	**Riyadh** mean±SD Median (IQR)		**Medina** mean±SD Median (IQR)		**Dammam** mean±SD Median (IQR)		**Al Ahsa** mean±SD Median (IQR)	
Day	<135 mmol/L	135-145 mmol/L	<135 mmol/L	135-145 mmol/L	<135 mmol/L	135-145 mmol/L	<135 mmol/L	135-145 mmol/L
Sunday	7.47±5.85 5 (3-10)	6.98±5.91 5 (3-9)	7.31±5.58 5 (4-9)	6.2±5.64 4 (3-8)	6.36±5.04 5 (3-8)	5.12±4.04 4 (3-6)	7.45±5.57 5 (3.5-10)	6.6±5.27 5 (3-9)
Monday	7.6±6.09 5 (3-10)	6.86±5.8 5 (3-9)	6.58±4.86 5 (3-9)	6.86±5.86 4 (3-9)	6±4.34 5 (3-8)	4.53±3.14 3 (2-5)	7.38±4.94 6 (4-9)	6.64±4.9 4 (3-9)
Tuesday	8.08±6.09 6 (4-10)	7.35±5.76 5 (3-10)	7.05±5.7 6 (3-9)	6.8±5.65 5 (3-9)	6.32±3.93 5 (4-9)	5.52±4.5 4 (3-7)	8.28±6.07 7 (4-10)	6.45±5.28 4 (3-8)
Wednesday	7.77±5.87 6 (3-10)	7.34±6.14 6 (3-9)	8.37±6.63 6 (4-10)	7.47±6.2 6 (3-10)	6.82±5.61 6 (3-8)	4.72±3.52 4 (2-6)	7.39±5.13 6 (3-9)	6.65±5.87 5 (3-8)
Thursday	7.85±5.9 6 (4-10)	7.76±6.22 6 (3-10)	8.38±6.08 6 (4-12)	7.8±5.63 6 (4-11)	6.39±4.63 5 (3-8)	6.1±5.39 5 (3-6.25)	8.2±5.97 6 (5-10)	6.46±5.04 5 (3-8)
Friday	7.83±5.8 6 (4-10.5)	7.98±5.94 6 (4-11)	8.33±5.93 6 (4-12)	7.43±5.71 6 (3-9)	7.61±5.68 6 (4-8)	6.44±5.75 5 (3-7)	8.45±6.3 6 (4-11)	7.1±4.77 6 (4-8)
Saturday	7.78±5.77 6 (4-11)	7.34±5.7 5 (3-10)	7.97±6.01 6 (3-11)	6.47±5.13 5 (3-8)	6.97±5.31 6 (3-8.5)	5.05±4.14 4 (3-6)	7.43±5.09 6 (4-10)	7.24±5.68 5 (3-9)
Total	7.76±5.92 6 (4-10)	7.35±5.94 5 (3-9)	7.67±5.84 6 (4-10)	6.99±5.72 5 (3-9)	6.63±4.98 5 (3-8)	5.31±4.41 4 (3-6)	7.79±5.61 6 (4-10)	6.7±5.29 5 (3-8)

Legend to Table S3. This table compares the length of stay of admissions with hyponatremia (serum sodium <135 mmol/l) with that of admissions who were normonatremic (135-145 mmol/L). Data are stratified by day of admission and hospital site. Data are presented as mean ± standard deviation (SD) and median (interquartile range; IQR).
