# Supplementary material for: Associations between day of admission, admission hyponatremia and hospital outcomes in medical patients: A retrospective multicenter cohort study
Source: PLoS One. 2025 Oct 27;20(10):e0335248. doi: 10.1371/journal.pone.0335248 (PMC12558553; doi:10.1371/journal.pone.0335248)
Supplement: S4 Table — Legend. A chi-squared test demonstrated statistically significant association between the day of admission and the numbers of normonatremic medical admissions transferred to an intensive therapy unit (ITU; p < 0.00000001). This finding indicates that the distribution of ITU admissions varies significantly with the day of admission, rather than establishing a causal link. In Saudi Arabia the weekend is Friday-Saturday, while Sunday to Thursday are weekdays. Serial post hoc testing with pairwise Chi-squared tests is shown. Statistically significant differences (p < 0.05) are indicated (*). (PDF) [file pone.0335248.s004.pdf]

Appendix Table S4. The associations of admission day with ITU admissions in normonatremic patients

Day	Sunday	Monday	Tuesday	Wednesday	Thursday	Friday	Saturday
**N; % (95%CI)**	689; 20.9% (19.5% to 22.3%)	693; 20.5% (19.1% to 21.8%)	707; 21.2% (19.8% to 22.6%)	721; 22.1% (20.7% to 23.5%)	699; 24% (22.5% to 25.6%)	640; 25.1% (23.4% to 26.8%)	691; 26.5% (24.8% to 28.1%)
**Sun**	1	0.66	0.79	0.25	0.0037*	0.0015*	0.02*
**Mon**		1	0.48	0.11	0.00079*	0.000024*	0.000000054*
**Tue**			1	0.37	0.0078*	0.00039*	0.0000020*
**Wed**				1	0.075	0.0071*	0.0001*
**Thu**					1	0.34	0.036*
**Fri**						1	0.27
**Sat**							1

Legend to Table S4. A chi-squared test demonstrated statistically significant association between the day of admission and the numbers of normonatremic medical admissions transferred to an intensive therapy unit (ITU; p<0.00000001). This finding indicates that the distribution of ITU admissions varies significantly with the day of admission, rather than establishing a causal link. In Saudi Arabia the weekend is Friday-Saturday, while Sunday to Thursday are weekdays. Serial post hoc testing with pairwise Chi-squared tests is shown. Statistically significant differences (p<0.05) are indicated (*).
