# Supplementary material for: Associations between day of admission, admission hyponatremia and hospital outcomes in medical patients: A retrospective multicenter cohort study
Source: PLoS One. 2025 Oct 27;20(10):e0335248. doi: 10.1371/journal.pone.0335248 (PMC12558553; doi:10.1371/journal.pone.0335248)
Supplement: S5 Table — Legend. A chi-squared test demonstrated statistically significant association between the day of admission and the numbers of hyponatremic medical admissions transferred to an intensive therapy unit (ITU; p = 0.0036). This finding indicates that the distribution of ITU admissions in this cohort varied significantly with the day of admission, rather than establishing a causal link. In Saudi Arabia the weekend is Friday-Saturday, while Sunday to Thursday are weekdays. Serial post hoc testing with pairwise Chi-squared test is shown. Statistically significant differences (p < 0.05) are indicated (*). (PDF) [file pone.0335248.s005.pdf]

**Appendix Table S5. The associations of admission day with ITU admissions in hyponatremic patients**

| Day | Sunday | Monday | Tuesday | Wednesday | Thursday | Friday | Saturday |
|---|---|---|---|---|---|---|---|
| **N; % (95%CI)** | 576; 22% (20.4% - 23.6%) | 564; 22.3% (20.6% - 23.9%) | 555; 23% (21.3% - 24.7%) | 534; 21.5% (19.9% - 23.1%) | 587; 24.3% (22.6% - 26%) | 492; 21.6% (20% - 23.3%) | 614; 25.6% (23.9% - 27.4%) |
| **Sun** | 1 | 0.29 | 0.93 | 0.98 | 0.29 | 0.84 | 0.15 |
| **Mon** | | 1 | 0.69 | 0.63 | 0.020* | 0.49 | 0.015* |
| **Tue** | | | 1 | 0.98 | 0.81 | 0.87 | 0.36 |
| **Wed** | | | | 1 | 0.75 | 0.87 | 0.39 |
| **Thu** | | | | | 1 | 0.40 | 0.87 |
| **Fri** | | | | | | 1 | 0.54 |
| **Sat** | | | | | | | 1 |

Legend to Table S5. A chi-squared test demonstrated statistically significant association between the day of admission and the numbers of hyponatremic medical admissions transferred to an intensive therapy unit (ITU; p=0.0036). This finding indicates that the distribution of ITU admissions varies significantly with the day of admission, rather than establishing a causal link. In Saudi Arabia the weekend is Friday-Saturday, while Sunday to Thursday are weekdays. Serial post hoc testing with pairwise Chi-squared test is shown. Statistically significant differences (p<0.05) are indicated (*).
