# Supplementary material for: Associations between day of admission, admission hyponatremia and hospital outcomes in medical patients: A retrospective multicenter cohort study
Source: PLoS One. 2025 Oct 27;20(10):e0335248. doi: 10.1371/journal.pone.0335248 (PMC12558553; doi:10.1371/journal.pone.0335248)
Supplement: S6 Table — Legend. This table presents the association between day of admission and the length of stay (LOS) of admissions with normonatremia. The LOS is presented as Mean± standard deviation (SD) and Median (interquartile range (IQR)). The Kruskal-Wallis test demonstrated significant association of admission day with the LOS of normonatremic medical inpatients (p = 1.28E-31), indicating that LOS varies significantly based on the day of admission. This observation does not imply a causal relationship. Post hoc testing with Dunn’s test is shown. Statistically significant differences are indicated (*). (PDF) [file pone.0335248.s006.pdf]

Appendix Table S6. Association between length of stay of normonatremic patients and admission day

Day	Sunday	Monday	Tuesday	Wednesday	Thursday	Friday	Saturday
**LOS Days Mean±SD Median (IQR)**	7.41±5.76 5 (3-10)	7.41±5.82 5 (3-10)	7.93±6 6 (3-10)	7.76±5.87 6 (3-9)	7.88±5.88 6 (4-10)	7.94±5.87 6 (4-11)	7.73±5.72 6 (4-11)
**Sunday**	1	1	0.14	0.074	1.00×10^{-6}*	4.31×10^{-10}*	0.27
**Monday**		1	0.085	0.042*	3.01×10^{-7}*	1.02×10^{-10}*	0.19
**Tuesday**			1	1	0.041*	0.00027*	1
**Wednesday**				1	0.089	0.00086*	1
**Thursday**					1	0.79	0.045*
**Friday**						1	0.0004*
**Saturday**							1

Legend to Table S6. This table presents the association between day of admission and the length of stay (LOS) of admissions with normonatremia. The LOS is presented as Mean± standard deviation (SD) and Median (interquartile range (IQR)). The Kruskal-Wallis test demonstrated significant association of admission day with the LOS of normonatremic medical admission episodes ($p=1.28 \times 10^{-31}$), indicating that LOS varies significantly based on the day of admission. This observation does not imply a causal relationship. Post hoc testing with Dunn's test is shown. Statistically significant differences are indicated (*).
