# Supplementary material for: Associations between day of admission, admission hyponatremia and hospital outcomes in medical patients: A retrospective multicenter cohort study
Source: PLoS One. 2025 Oct 27;20(10):e0335248. doi: 10.1371/journal.pone.0335248 (PMC12558553; doi:10.1371/journal.pone.0335248)
Supplement: S7 Table — Legend. This table shows the association between admission day and the length of stay (LOS) of admissions with hyponatremia. The LOS is presented as mean±standard deviation (SD) and median (interquartile range (IQR)). The Kruskal-Wallis test demonstrated a statistically significant association between day of admission and the LOS of hyponatremic medical admissions (p = 5.48x10^-9^). This observation does not imply a causal relationship. Post hoc testing with Dunn’s test is shown. Statistically significant differences are indicated (*). (PDF) [file pone.0335248.s007.pdf]

Appendix Table S7. Association between length of stay of hyponatremic patients and admission day

Day	Sunday	Monday	Tuesday	Wednesday	Thursday	Friday	Saturday
**LOS Days Mean±SD Median (IQR)**	6.74±5.74 5 (3-9)	6.72±5.64 4 (3-9)	7.11±5.66 5 (3-9)	7.14±6.04 5 (3-9)	7.55±6.04 6 (3-9)	7.77±5.83 6 (4-11)	7.08±5.58 5 (3-9)
**Sunday**	1	0.91	0.00018*	0.0013*	0.0000047*	0.0000013*	0.0025
**Monday**		1	0.00013*	0.00093*	0.0000032*	0.00000086*	0.0018*
**Tuesday**			1	0.59	0.41	0.25	0.49
**Wednesday**				1	0.17	0.094	0.87
**Thursday**					1	0.74	0.13
**Friday**						1	0.068
**Saturday**							1

Legend to Table S7. This table shows the association between admission day and the length of stay (LOS) of admissions with hyponatremia. The LOS is presented as mean±standard deviation (SD) and median (interquartile range (IQR)). The Kruskal-Wallis test demonstrated a statistically significant association between day of admission and the LOS of hyponatremic medical admission episodes ($p=5.48x10^{-9}$). This observation does not imply a causal relationship. Post hoc testing with Dunn's test is shown. Statistically significant differences are indicated (*).
