# Supplementary material for: Associations between day of admission, admission hyponatremia and hospital outcomes in medical patients: A retrospective multicenter cohort study
Source: PLoS One. 2025 Oct 27;20(10):e0335248. doi: 10.1371/journal.pone.0335248 (PMC12558553; doi:10.1371/journal.pone.0335248)
Supplement: S8 Table — Legend. This table shows the association between admission day and the length of stay (LOS) of admissions with mild hyponatremia. The LOS is presented as Mean± standard deviation (SD) and Median (interquartile range (IQR)). The Kruskal-Wallis test demonstrated statistically significant association between admission day and the LOS of mildly hyponatremic medical inpatients (serum sodium 130–134.9 mmol/L; p = 5.48x10^-9^). Post hoc testing with Dunn’s test is shown. These tests do not imply a causal relationship. Statistically significant differences are indicated (*). (PDF) [file pone.0335248.s008.pdf]

Appendix Table S8. Association between length of stay and admission day in mildly hyponatremic patients

Day	Sunday	Monday	Tuesday	Wednesday	Thursday	Friday	Saturday
**LOS Days Mean±SD Median (IQR)**	7.1 ± 5.6 5 (3-9)	8 ± 6.1 6 (3-10)	7.7 ± 5.9 6 (3-10)	7.6 ± 5.7 6 (4-9)	8 ± 6.1 6 (4-11)	7.6 ± 5.8 6 (4-10)	7.2 ± 5.8 5 (3-9)
**Sunday**	1	0.80	0.00029*	0.0023*	0.0027	$8.61x10^{-6}$*	0.018*
**Monday**		1	0.00012*	0.0010*	0.0013*	$3.22x10^{-6}$*	0.0096*
**Tuesday**			1	0.55	0.59	0.35	0.25
**Wednesday**				1	0.97	0.13	0.56
**Thursday**					1	0.15	0.55
**Friday**						1	0.042*
**Saturday**							1

Legend to Table S8. This table shows the association between admission day and the length of stay (LOS) of admissions with mild hyponatremia. The LOS is presented as Mean± standard deviation (SD) and Median (interquartile range (IQR)). The Kruskal-Wallis test demonstrated statistically significant association between admission day and the LOS of mildly hyponatremic medical admission episodes (serum sodium 130-134.9 mmol/L; $p= 5.48x10^{-9}$). Post hoc testing with Dunn's test is shown. These tests do not imply a causal relationship. Statistically significant differences are indicated (*).
