# Supplementary material for: Associations between day of admission, admission hyponatremia and hospital outcomes in medical patients: A retrospective multicenter cohort study
Source: PLoS One. 2025 Oct 27;20(10):e0335248. doi: 10.1371/journal.pone.0335248 (PMC12558553; doi:10.1371/journal.pone.0335248)
Supplement: S9 Table — Legend. This table shows the association between admission day and the length of stay (LOS) of admissions with moderate hyponatremia. The LOS is presented as Mean± standard deviation (SD) and Median (interquartile range (IQR)). The Kruskal-Wallis test demonstrated a statistically significant association between the day of admission and the LOS of moderately hyponatremic medical admissions (serum sodium 125–129.9 mmol/L; p = 5.48x10^-9^). Post hoc testing with Dunn’s test is shown. Statistically significant differences are indicated (*). (PDF) [file pone.0335248.s009.pdf]

Appendix Table S9. Association between length of stay and admission day in moderately hyponatremic patients

Day	Sunday	Monday	Tuesday	Wednesday	Thursday	Friday	Saturday
**LOS Days**<br>**Mean±SD**<br>**Median (IQR)**	7.7 ± 5.8<br>5 (3-10)	7.7 ± 6.2<br>5 (3-10)	8.1 ± 5.9<br>7 (4-10)	8.2 ± 6.2<br>7 (4-10)	8.7 ± 6.2<br>7 (4-12)	7.9 ± 5.6<br>6 (4-11)	8 ± 5.9<br>6 (4-11)
**Sunday**	1	0.65	0.13	0.20	0.00056*	0.17	0.14
**Monday**		1	0.058	0.042*	0.00016*	0.078	0.063
**Tuesday**			1	0.93	0.075	0.90	0.91
**Wednesday**				1	0.082	0.83	0.84
**Thursday**					1	0.057	0.049*
**Friday**						1	0.98
**Saturday**							1

Legend to Table S9. This table shows the association between admission day and the length of stay (LOS) of admissions with moderate hyponatremia. The LOS is presented as Mean± standard deviation (SD) and Median (interquartile range (IQR)). The Kruskal-Wallis test demonstrated a statistically significant association between the day of admission and the LOS of moderately hyponatremic medical admission episodes (serum sodium 125-129.9 mmol/L; p= 5.48×10^{-9}). Post hoc testing with Dunn's test is shown. Statistically significant differences are indicated (*).
