# Supplementary material for: Associations between day of admission, admission hyponatremia and hospital outcomes in medical patients: A retrospective multicenter cohort study
Source: PLoS One. 2025 Oct 27;20(10):e0335248. doi: 10.1371/journal.pone.0335248 (PMC12558553; doi:10.1371/journal.pone.0335248)
Supplement: S10 Table — Legend. This table shows the association between admission day and the length of stay (LOS) of admissions with severe hyponatremia. The LOS is presented as mean± standard deviation (SD) and median (interquartile range (IQR)). The Kruskal-Wallis test demonstrated a statistically significant association between the day of admission and the LOS of severely hyponatremic medical admissions (serum sodium < 125 mmol/L; (p = 0.88). Post hoc testing with Dunn’s test is shown. There were no statistically significant differences (p < 0.05). (PDF) [file pone.0335248.s010.pdf]

Appendix Table S10. Association between length of stay and admission day in severely hyponatremic patients

Day	Sunday	Monday	Tuesday	Wednesday	Thursday	Friday	Saturday
**LOS Days Mean±SD Median (IQR)**	7.6 ± 5.7 5 (4-10)	7.9 ± 6.1 5 (4-9.25)	7.8 ± 5.8 6 (4-9)	7.4 ± 5.5 6 (4-9)	7.8 ± 5.9 5 (4-9)	7.8 ± 5.6 6 (4-9)	7.7 ± 5.2 6 (4-10.75)
**Sunday**	1	0.66	0.59	0.85	0.30	0.12	0.44
**Monday**		1	0.92	0.81	0.55	0.26	0.73
**Tuesday**			1	0.74	0.63	0.32	0.82
**Wednesday**				1	0.41	0.18	0.57
**Thursday**					1	0.60	0.79
**Friday**						1	0.43
**Saturday**							1

Legend to Table S10. This table shows the association between admission day and the length of stay (LOS) of admissions with severe hyponatremia. The LOS is presented as mean± standard deviation (SD) and median (interquartile range (IQR)). The Kruskal-Wallis test demonstrated a statistically significant association between the day of admission and the LOS of severely hyponatremic medical admission episodes (serum sodium <125 mmol/L; (p=0.88). Post hoc testing with Dunn's test is shown. There were no statistically significant differences (p<0.05).
