# Supplementary material for: Associations between day of admission, admission hyponatremia and hospital outcomes in medical patients: A retrospective multicenter cohort study
Source: PLoS One. 2025 Oct 27;20(10):e0335248. doi: 10.1371/journal.pone.0335248 (PMC12558553; doi:10.1371/journal.pone.0335248)
Supplement: S11 Table — Legend. This table shows the variation in serum sodium concentration with admission day. In Saudi Arabia the weekend is Friday-Saturday, while Sunday to Thursday are weekdays. Data presented as mean ± standard deviation (SD) and median and interquartile range (IQR). The differences were compared using the Kruskal-Wallis test. This revealed statistically significant differences in distribution of serum sodium between at least two days (H(6)=54.9, p = 4.41x10^-10^). Serial post hoc testing with Dunn’s test is shown. Statistically significant differences are indicated (*). (PDF) [file pone.0335248.s011.pdf]

Appendix Table S11. The variation in serum sodium concentration with day of admission

Day	Sunday	Monday	Tuesday	Wednesday	Thursday	Friday	Saturday
**Sodium mmol/L mean±SD Median (IQR)**	134.8 ± 7.6<br>135 (132-138)	134.8 ± 7.6<br>136 (132-138)	134.9 ± 7.5<br>136 (132-138)	134.8 ± 7.6<br>135 (132-138)	134.3 ± 7.5<br>135 (131-138)	134.4 ± 8.2<br>135 (131-138)	134.3 ± 8.1<br>135 (131-138)
**Sunday**	1	0.22	0.024*	0.26	0.047*	0.027*	0.00024*
**Monday**		1	0.29	0.94	0.0015*	0.00075*	1.27×10^{-6}*
**Tuesday**			1	0.26	3.05×10^{-5}*	1.39×10^{-5}*	5.91×10^{-9}*
**Wednesday**				1	0.0021*	0.0011*	2.13×10^{-6}*
**Thursday**					1	0.79	0.094
**Friday**						1	0.17
**Saturday**							1

Legend to Table S11. This table shows the variation in serum sodium concentration with admission day. In Saudi Arabia the weekend is Friday-Saturday, while Sunday to Thursday are weekdays. Data presented as mean ± standard deviation (SD) and median and interquartile range (IQR). The differences were compared using the Kruskal-Wallis test. This revealed statistically significant differences in the distribution of serum sodium concentrations between at least two days ($H(6)=54.9$, $p=4.41 \times 10^{-10}$). Serial post hoc testing with Dunn's test is shown. Statistically significant differences are indicated (*).
