# Supplementary material for: Associations between day of admission, admission hyponatremia and hospital outcomes in medical patients: A retrospective multicenter cohort study
Source: PLoS One. 2025 Oct 27;20(10):e0335248. doi: 10.1371/journal.pone.0335248 (PMC12558553; doi:10.1371/journal.pone.0335248)
Supplement: S12 Table — Legend. This table shows the variation in severity of hyponatremia with admission day. In Saudi Arabia the weekend is Friday-Saturday, while Sunday to Thursday are weekdays. The differences were compared using Chi-squared tests. This revealed statistically significant differences in the severity of hyponatremia between at least two days. Serial post hoc testing with pairwise chi-squared tests is shown. Statistically significant differences after the application of Bonferroni correction (p < 0.0071; i.e., 0.05/7) are indicated (*). (PDF) [file pone.0335248.s012.pdf]

Appendix Table S12. The variation in severity of hyponatremia with day of admission

Day	Sunday	Monday	Tuesday	Wednesday	Thursday	Friday	Saturday
**Sunday**	1	0.038	0.0076	0.48	0.041	8.05×10^{-5}*	2.68×10^{-5}*
**Monday**		1	0.53	0.16	0.007*	0.00004*	1.20×10^{-7}*
**Tuesday**			1	0.20	6.93×10^{-8}*	6.93×10^{-8}*	4.3×10^{-11}*
**Wednesday**				1	1.73×10^{-6}*	1.73×10^{-6}*	3.92×10^{-8}*
**Thursday**					1	0.09	0.077
**Friday**						1	0.24
**Saturday**							1

Legend to Table S12. This table shows the variation in severity of hyponatremia with admission day. In Saudi Arabia the weekend is Friday-Saturday, while Sunday to Thursday are weekdays. The differences were compared using Chi-squared tests. This revealed statistically significant differences in the severity of hyponatremia between at least two days. Serial post hoc testing with pairwise chi-squared tests is shown. Statistically significant differences after the application of Bonferroni correction ($p<0.0071$; i.e. 0.05/7) are indicated (*).
