# Supplementary material for: Associations between day of admission, admission hyponatremia and hospital outcomes in medical patients: A retrospective multicenter cohort study
Source: PLoS One. 2025 Oct 27;20(10):e0335248. doi: 10.1371/journal.pone.0335248 (PMC12558553; doi:10.1371/journal.pone.0335248)
Supplement: S13 Table — Legend. This table shows the variation in the number of admissions before the next weekend stratified by serum sodium concentration and admission day. In Saudi Arabia the weekend is Friday-Saturday, while Sunday to Thursday are weekdays. The differences were compared using Chi-squared tests. This revealed statistically significant differences in the percentages of admission episodes discharged before the next weekend between at least two days. Serial post hoc testing with pairwise chi-squared tests is shown. Statistically significant differences after the application of Bonferroni correction (p < 0.0071; i.e., 0.05/7) are indicated (*). (PDF) [file pone.0335248.s013.pdf]

Appendix Table S13. The patients discharged before the next weekend stratified by day of admission and serum sodium

Day		Patients discharged before the next weekend N; % (95%CI)		
Serum Sodium		Hyponatremia (<135 mmol/L)	Normonatremia (135-145 mmol/L)	p
Weekend	Friday	1439, 63.3% (61.3%-65.3%)	1652, 64.8% (62.9%-66.7%)	0.28
	Saturday	1390, 58% (56%-60%)	1639, 62.7% (60.8%-64.6%)	0.00062*
Weekday	Sunday	1418, 54.2% (52.3%-56.1%)	1964, 59.6% (57.9%-61.3%)	3.14×10^{-5}*
	Monday	1072, 42.3% (40.4%-44.2%)	1692, 50% (48.3%-51.7%)	4.39×10^{-9}*
	Tuesday	606, 25.1% (23.4%-26.8%)	1066, 31.9% (30.3%-33.5%)	2.14×10^{-8}*
	Wednesday	274, 11% (9.8%-12.2%)	589, 18.1% (16.8%-19.4%)	1.51×10^{-13}*
	Thursday	48, 2% (1.4%-2.6%)	155, 5.3% (4.5%-6.1%)	2.51×10^{-10}*
Total		6247, 36.5% (35.8%-37.2%)	8757, 41% (40.3%-41.7%)	

Legend to Table S13. This table shows the variation in the number of admissions discharged before the next weekend stratified by serum sodium concentration and admission day. In Saudi Arabia the weekend is Friday-Saturday, while Sunday to Thursday are weekdays. The differences were compared using Chi-squared tests. This revealed statistically significant differences in the percentages of admission episodes discharged before the next weekend between at least two days. Serial post hoc testing with pairwise chi-squared tests is shown. Statistically significant differences after the application of Bonferroni correction (p<0.0071; i.e. 0.05/7) are indicated (*). These data are also presented in Fig S1.
