# Supplementary material for: Associations between day of admission, admission hyponatremia and hospital outcomes in medical patients: A retrospective multicenter cohort study
Source: PLoS One. 2025 Oct 27;20(10):e0335248. doi: 10.1371/journal.pone.0335248 (PMC12558553; doi:10.1371/journal.pone.0335248)
Supplement: S14 Table — Legend. This table presents the demographics, serum sodium, intensive therapy unit (ITU) admissions and outcomes (length of stay, and mortality) of the study population stratified by admission before (pre-COVID-19) or during the COVID-19 period. Data are presented as mean ± standard deviation (SD) or frequency and percentages (%) as appropriate. (PDF) [file pone.0335248.s014.pdf]

Appendix Table S14. Demographics, serum sodium on admission and outcomes of the whole study population stratified by admission before or during COVID-19 pandemic period.

	Pre-COVID	COVID-19
**DEMOGRAPHICS**		
**Total Admissions (N%)**	25,401 (62.9%)	14,965 (37.1%)
**Female (N%)**	12,839 (51%)	7,555 (50%)
**Male (N%)**	12,562 (49%)	7,410 (50%)
**Age Years (mean±SD)**	62.2 ± 20.1	62.5 ± 19.9
**SERUM SODIUM CONCENTRATION ON ADMISSION (N=40366)**		
**<135 mmol/L (N%)**	13,275 (52%)	8,074 (54%)
**135–145 mmol/L (N%)**	11008 (43.3%)	6117 (40.9%)
**>145 mmol/L (N%)**	1,118 (4.4%)	774 (5.2%)
**Sodium mmol/L (mean±SD)**	134.5 ± 7.6	134.9 ± 8.0
**OUTCOMES**		
**Length of stay Days (mean±SD)**	7.55 ± 5.9	7.49 ± 5.94
**ITU admission (N%)**	5,802 (23%)	3,466 (23%)
**Mortality (N%)**	1,346 (5.3%)	1,082 (7.2%)

Legend to Table S14. This table presents the demographics, serum sodium, intensive therapy unit (ITU) admissions and outcomes (length of stay, and mortality) of the study population stratified by admission before (pre-COVID-19) or during the COVID-19 period. Data are presented as mean ± standard deviation (SD) or frequency and percentages (%) as appropriate.
