# Supplementary material for: Associations between day of admission, admission hyponatremia and hospital outcomes in medical patients: A retrospective multicenter cohort study
Source: PLoS One. 2025 Oct 27;20(10):e0335248. doi: 10.1371/journal.pone.0335248 (PMC12558553; doi:10.1371/journal.pone.0335248)
Supplement: S15 Table — Legend. Results of multivariable regression analysis examining the association between age, sex, admission day, serum sodium and admission to the intensive therapy unit (ITU), mortality or length of stay (LOS). Three models were constructed: logistic regression for intensive therapy unit (ITU) admission and inpatient mortality (presented as adjusted odds ratios (OR) with 95% Confidence Intervals (CI)) and gamma regression for LOS (presented as incidence rate ratios (IRR) with 95%CI). These analyses serve as a sensitivity analysis to assess the impact of the COVID-19 period on these outcomes and how it interacts with other predictors. Dummy coding (one-hot encoding) was used for day-of-week variables using Sunday as the reference. In Saudi Arabia the weekend is Friday-Saturday, while Sunday to Thursday are weekdays. Male was the reference for sex. The pre-COVID period was the reference for the COVID period. P-values are based on two-tailed tests. Note: Model fit statistics differ from Table 4 due to inclusion of interaction terms. Model fit statistics: Mortality model (N = 38,474; AIC = 12,863; BIC = 13,042). ITU Admission model (N = 38,474; AIC = 39,545; BIC = 39,716). LOS model (N = 38,474; AIC = 215,775; BIC = 215,963). Abbreviations: AIC, Akaike Information Criterion; BIC, Bayesian Information Criterion; N, Number of Observations. (PDF) [file pone.0335248.s015.pdf]

Appendix Table S15. Multivariable Regression Models for Inpatient Mortality, ITU Admission, and Length of Stay (LOS): Sensitivity Analysis for COVID Period vs. Pre-COVID

	ITU Admission		Mortality		Length of Stay	
**Variable**	**Adjusted OR (95%CI)**	**p-value**	**Adjusted OR (95%CI)**	**p-value**	**Adjusted IRR (95%CI)**	**p-value**
**Age**	1.00 (1.00 to 1.00)	0.015	1.04 (1.03 to 1.04)	<0.001	1.00 (1.00 to 1.00)	<0.001
**Sex**						
Male	—		—		—	
Female	0.87 (0.83 to 0.92)	<0.001	0.90 (0.82 to 0.99)	0.030	1.00 (0.99 to 1.01)	0.584
**Admission day**						
Sun	—		—		—	
Mon	1.05 (0.94 to 1.18)	0.382	1.03 (0.82 to 1.30)	0.793	1.00 (0.97 to 1.03)	0.945
Tue	1.03 (0.92 to 1.16)	0.590	0.91 (0.72 to 1.15)	0.430	1.04 (1.00 to 1.07)	0.028
Wed	1.07 (0.96 to 1.20)	0.223	0.93 (0.73 to 1.17)	0.528	1.03 (0.99 to 1.06)	0.124
Thu	1.20 (1.07 to 1.34)	0.002	1.11 (0.88 to 1.39)	0.391	1.08 (1.04 to 1.11)	<0.001
Fri	1.18 (1.04 to 1.32)	0.008	0.99 (0.78 to 1.26)	0.943	1.08 (1.04 to 1.12)	<0.001
Sat	1.38 (1.23 to 1.55)	0.000	1.02 (0.80 to 1.28)	0.897	0.99 (0.96 to 1.03)	0.652
**COVID**						
Pre-COVID	—		—		—	
COVID	1.00 (0.87 to 1.14)	0.971	1.74 (1.37 to 2.21)	0.000	0.96 (0.93 to 1.00)	0.057
**Admission Day x COVID Period**						
Sun	—					
Mon	0.88 (0.73 to 1.06)	0.165	0.69 (0.48 to 0.98)	0.037	1.00 (0.94 to 1.05)	0.868
Tue	1.03 (0.86 to 1.25)	0.733	0.83 (0.58 to 1.18)	0.303	1.04 (0.99to 1.05)	0.141
Wed	0.95 (0.79 to 1.15)	0.617	0.98 (0.69 to 1.38)	0.905	1.04 (0.98 to 1.10)	0.196
Thu	1.03 (0.85 to 1.24)	0.774	0.59 (0.42 to 0.84)	0.004	1.00 (0.95 to 1.06)	0.982
Fri	0.98 (0.81 to 1.19)	0.844	0.74 (0.52 to 1.07)	0.108	1.04 (0.98 to 1.11)	0.157
Sat	0.91 (0.75 to 1.10)	0.323	0.89 (0.63 to 1.26)	0.524	1.05 (0.99 to 1.11)	0.115

**ITU admission**						
No	—	—	—		—	
Yes	—	—	11.82 (10.66 to 13.12)	<0.001	1.73 (1.70 to 1.77)	<0.001
**Hospital**						
Riyadh	—		—		—	
Medina	1.91 (1.78 to 2.06)	<0.001	0.80 (0.69 to 0.92)	0.003	0.91 (0.88 to 0.93)	<0.001
Dammam	2.17 (1.96 to 2.41)	<0.001	0.41 (0.30 to 0.54)	<0.001	0.74 (0.72 to 0.77)	<0.001
Al Ahsa	4.20 (3.91 to 4.51)	<0.001	0.71 (0.61 to 0.82)	<0.001	0.84 (0.82 to 0.86)	<0.001
**Serum sodium (per mmol/l)**	1.00 (1.00 to 1.01)	0.166	1.01 (1.00 to 1.02)	0.002	1.00 (1.00 to 1.00)	0.005
**Intercept**	0.13 (0.08 to 0.23)	<0.001	0.00 (0.00 to 0.00)	<0.001	5.37 (4.57 to 6.33)	<0.001

Legend to Table S15. Results of multivariable regression analysis examining the association between age, sex, admission day, serum sodium and admission to the intensive therapy unit (ITU), mortality or length of stay (LOS). Three models were constructed: logistic regression for intensive therapy unit (ITU) admission and inpatient mortality (presented as adjusted odds ratios (OR) with 95% Confidence Intervals (CI)) and gamma regression for LOS (presented as incidence rate ratios (IRR) with 95%CI). These analyses serve as a sensitivity analysis to assess the impact of the COVID-19 period on these outcomes and how it interacts with other predictors. Dummy coding (one-hot encoding) was used for day-of-week variables using Sunday as the reference. In Saudi Arabia the weekend is Friday-Saturday, while Sunday to Thursday are weekdays. Male was the reference for sex. The pre-COVID period was the reference for the COVID period. P-values are based on two-tailed tests. Note: Model fit statistics differ from Table 4 due to inclusion of interaction terms. Model fit statistics: Mortality model (N=38,474; AIC=12,863; BIC=13,042). ITU Admission model (N=38,474; AIC=39,545; BIC=39,716). LOS model (N=38,474; AIC=215,775; BIC=215,963). Abbreviations: AIC, Akaike Information Criterion; BIC, Bayesian Information Criterion; N, Number of Observations.
