# Supplementary figures and images for: Associations between day of admission, admission hyponatremia and hospital outcomes in medical patients: A retrospective multicenter cohort study
Source: PLoS One. 2025 Oct 27;20(10):e0335248. doi: 10.1371/journal.pone.0335248 (PMC12558553; doi:10.1371/journal.pone.0335248)

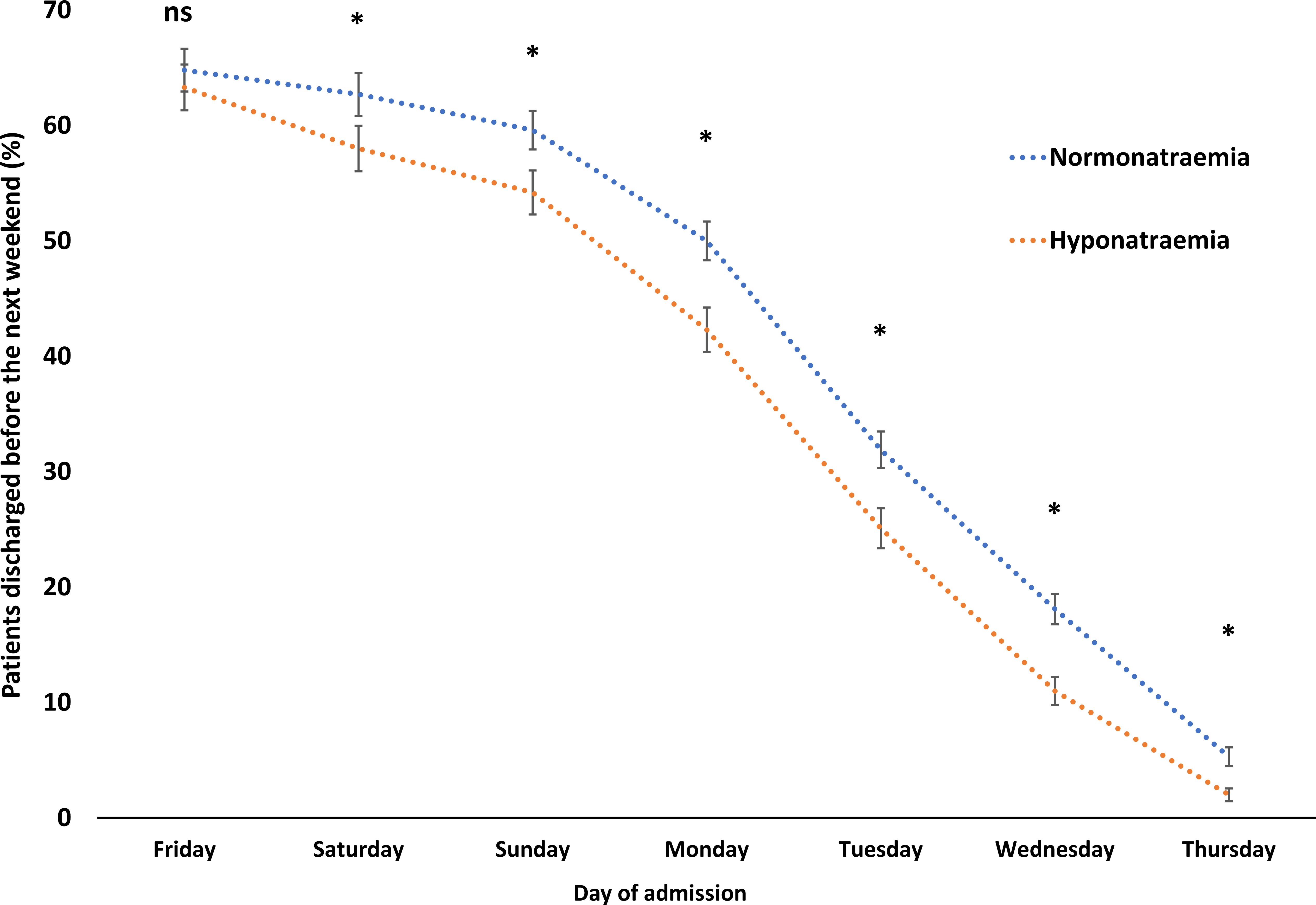

Supplement: S1 Fig — Legend. This figure shows the variation in the percentage of admission episodes discharged before the next weekend stratified by serum sodium concentration and admission day. In Saudi Arabia the weekend is Friday-Saturday, while Sunday to Thursday are weekdays. The differences were compared using Chi-squared tests. This revealed statistically significant differences in the percentages of admission episodes discharged before the next weekend between at least two days. Serial post hoc testing with pairwise chi-squared tests is shown. Statistically significant differences after the application of Bonferroni correction (p < 0.0071; i.e., 0.05/7) are indicated (*). (TIF) [file pone.0335248.s017.tif]

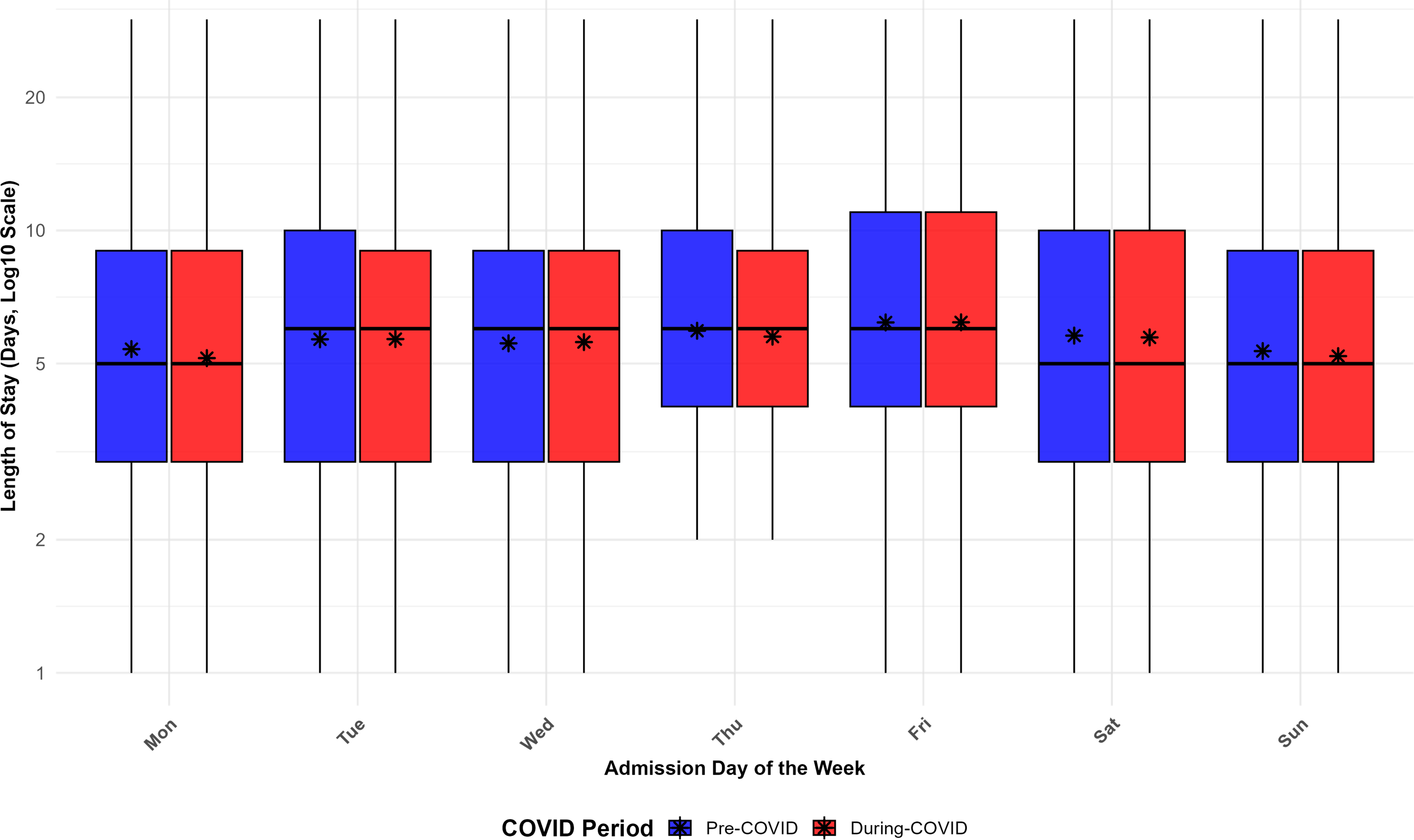

Supplement: S2 Fig — Legend. This Kaplan-Meier analysis assessed the 30-day inpatient survival of admissions before (01/01/16 to 29/02/20 (pre-COVID)) or during the COVID-19 period (01/03/20 to 31/05/22). A statistically significant increase in inpatient mortality during the COVID-19 pandemic period was observed (1082 (7.2%); pre-COVID 1346 (5.3%); log rank χ2 = 54.1 p = 2x10^-13^). No statistically significant differences were observed between groups (log-rank test, p = 0.15). 95% Confidence intervals are shown as shaded areas. (TIF) [file pone.0335248.s018.tif]

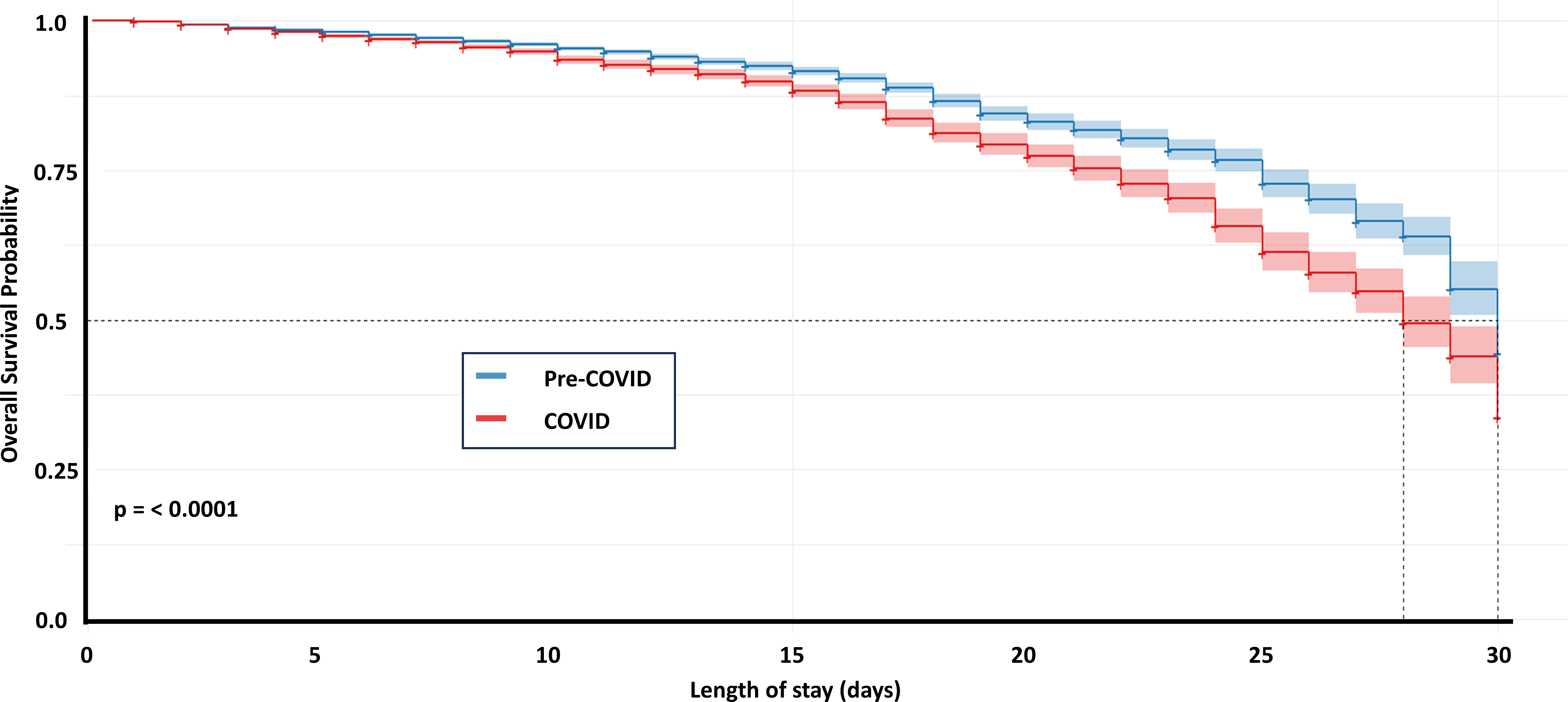

Supplement: S3 Fig — Legend. This box plot shows visually compares the length of hospital stay (in days, on a log10 scale) across different admission days, distinguishing between Pre-COVID (blue) and During-COVID (red) periods. Admissions with hypernatremia (serum sodium levels greater than 145 mmol/L) have been excluded from this analysis. Each box indicates the median LOS (horizontal line), the interquartile range (IQR) from the 25th to 75th percentile, and whiskers extending to data within 1.5 times the IQR (outliers are not displayed). The mean LOS is marked within each box (⋆). (TIF) [file pone.0335248.s019.tif]
